# Highly‐Conductive and Micro‐Structured Transparent Glass Substrates for Efficient and Scalable Photoelectrochemical Applications

**DOI:** 10.1002/advs.202515947

**Published:** 2026-04-09

**Authors:** Telmo da Silva Lopes, Jeffrey Capitão, Amin Khan, Leonardo Rodrigues, Dzmitry Ivanou, Tânia Lopes, Paula Dias, Adélio Mendes

**Affiliations:** ^1^ LEPABE – Laboratory for Process Engineering, Environment, Biotechnology and Energy ALiCE – Associate Laboratory in Chemical Engineering Faculty of Engineering University of Porto, Rua Dr. Roberto Frias Porto Portugal; ^2^ CONSTRUCT‐LFC Department of Civil Engineering Faculty of Engineering University of Porto Porto Portugal

**Keywords:** energy storage, hematite photoelectrodes, laser‐ablation lithography, photoelectrochemical cells, solar fuels, spray pyrolysis, transparent conductive oxides, upscaling

## Abstract

Photoelectrochemical (PEC) cells use semiconductor‐based photoelectrodes to directly convert sunlight into storable electrochemical fuels like hydrogen. Recent advances in material efficiency and stability have sparked interest in scaling this technology for industrial implementation. This work identifies key upscaling challenges that hinder the performance of large‐area PEC devices, specifically: (1) ohmic losses in the electron‐conductive substrate of the photoelectrode; (2) inhomogeneities of the photoabsorber; (3) ionic transport limitations in the electrolyte; (4) (photo)active area losses from bubble accumulation; and (5) concentration polarization and pH gradient losses at the photoelectrode–electrolyte interface. The first two challenges were addressed using a laser‐ablation lithography‐assisted spray pyrolysis procedure to produce uniform, conductive, stable, and high‐surface‐area transparent glass substrates. Micrometer‐thick fluorine‐doped tin oxide current collectors enhanced conductivity, while surface texturization maximized surface area. Device architecture (PortoCell), electrolyte concentration, and flow rate were optimized to mitigate the remaining limitations. The final optimized system, tested using a conformal hematite (α‐Fe_2_O_3_) thin film with *ca. *49 cm^2^ of photoactive area, displayed a photocurrent density of *ca. *0.63 mA cm^−2^ (1.45 V_RHE_, 100 mW cm^−2^); the same performance of a reference photoelectrode with *ca. *0.28 cm^2^. Furthermore, the proposed optimized large‐area photoelectrode demonstrated an operational long‐term stability of *ca. *1000 h.

## Graphical abstract

This work paves the way for practical photoelectrochemical water splitting devices by addressing key upscaling challenges related to substrate conductivity, absorber inhomogeneity, and mass transfer limitations. Integrated improvements in substrate engineering, material processing, device architecture, and operational conditions allow the development of a 49 cm^2^ PEC system (hematite photoelectrode) capable of replicating the performance of similar small‐area devices (<1 cm^2^) for >1000 h.

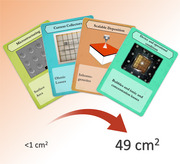

## 1 Introduction

Since the pioneering work by Fujishima and Honda [1], photoelectrochemical (PEC) cells have tempted the scientific community with the possibility of using renewable sunlight to provide society with crucial resources like electric power and chemical fuels. The latter have long been synthesized using indirect renewable energy‐driven electrolysis systems based on commercially available technologies. A prominent example is the so‐called PV‐electrolyser (PV‐EC) approach that uses PV cells to power a water electrolyser (an electrochemical—EC—device), thus storing solar energy in the form of green hydrogen (H_2_). Contrary to the PV‐EC approach, PEC cells offer a more integrated approach by coupling solar energy harvesting and chemical conversion within a single unit. This is typically accomplished by employing a photoactive semiconductor as at least one of the electrodes of an EC device, a component known as the photoelectrode [2]. The integration of the photoabsorber as a component of the EC device is traditionally performed in two distinct ways: (1) through a semiconductor‐liquid junction (SCLJ), where the semiconductor is in direct contact with the electrolyte; and (2) through a buried‐junction (BJ), where the photopotential of the device arises from internal solid‐state junctions independent‐of/protected‐from the electrolyte [3]. In both cases, operational current densities are in the range of tens of mA cm^−2^, two orders of magnitude below those observed for commercial electrolysers [4]. This unique feature decreases the influence of charge and mass transfer resistances on the performance of the device. As a result, less efficient but cheap earth‐abundant catalysts can become techno‐economically competitive [5, 6, 7].

Much of the research within the PEC field focuses on metal‐oxide semiconductors, a class of materials with a large variety of bandgaps, known for their abundance and compatibility with cost‐effective deposition techniques [8, 9, 10]. PEC‐PV tandem arrangements using metal‐oxide systems have consistently reached solar‐to‐hydrogen (*η*_STH_) conversion efficiencies above 7% (i.e., 8.1% for a WO_3_/BiVO_4_‐based system [11] and *ca. *7% for an α‐Fe_2_O_3_‐based photoelectrode [12]), and operational stability exceeding 1000 h [13, 14, 15]. Such developments provide a clear motivation to move for upscaled and more practical demonstrations. Strategic research programs, such as those from the United States Department of Energy (USDOE) [16] and the European Union Research and Innovation program (EUR&IP) [17], have set clear milestones for PEC‐WS: the US DOE targets 25% *η*_STH_, >200 h durability and >4 cm^2^ of active area, while the EUR&IP sets goals of >15% *η*_STH_, >500 h stability and in a device with at least 500 cm^2^ of active area.

The power output of PEC devices can be increased/scaled by either expanding the photoactive area or using solar concentration [18, 19, 20]. Scaling by photoactive area is the most common method for PEC‐WS devices [8] but remains underexplored. As an example, just slightly over 30 upscaled PEC‐WS demonstrations based on SCLJ systems have been developed so far (see Table S1– for an extensive overview) in a field with thousands of publications. Most works found in the literature are focused on increasing the efficiency and stability of photoabsorber materials, usually with very small photoactive areas (≤1 cm^2^). However, the balance of performance‐governing phenomena may severely change during upscale. Most demonstrations involving devices with photoactive areas above 25 cm^2^ display a fraction of the efficiency performance observed in similar small‐scale systems – Table 1. This indicates that upscaling PECs also requires a deep understanding of the multi‐physical (and often multi‐phase) transport phenomena occurring at the device level. As such, the architecture and relative positioning of all cell components, as well as the operating conditions, are pivotal if one wishes to optimize transport phenomena that appear especially relevant at larger scales (i.e., heat transfer, bubble formation, ionic and ohmic transport resistances) [18, 20, 21]. In addition, it is also essential to consider the compatibility of said components (including all electrode(s), membrane(s), embodiment, and sealants) with large‐area, high‐productivity, and low‐cost production techniques.

**TABLE 1:** Representative demonstrations of upscaled semiconductor‐liquid junction PEC‐WS ordered by publication date. The second‐to‐last column summarizes the upscale challenges addressed in the demonstrations (Challenge #1 – C1: Ohmic losses imposed by the substrate; Challenge #2 – C2: Inhomogeneity of photoabsorber layers; Challenge #3 – C3: Ionic losses and gas bubble formation/accumulation; Challenge #4 – C4: Losses from concentration polarization and pH gradients).

Area in small | large substrates^a^ / cm^2^	Photoelectrode (Substrate)	Photocurrent density in small | large substrates / mA cm^−2^	Photocurrent density loss with upscale/%	Operation time (of large‐area demo)	Upscale challenges addressed	Years, Refs.
0.36 | 130	WO_3_ (FTO‐coated glass with embedded Ag grid)	2.63 | ≈1.18 (@1.23 V_RHE_, AM 1.5G, 100 mW cm^−2^)	55	N/A	**C1**: use of line‐shaped Ag current collectors embedded on the FTO, protected by an epoxy resin; **C2**: scalable deposition techniques (screen‐printing); **C3** and **C4**: N/A	2011 [37]
<1 | 100	α‐Fe_2_O_3_ (FTO‐coated glass)	0.63b | 0.40 (@1.45 V_RHE_, AM 1.5G, 100 mW cm^−2^)	37	N/A	**C2**: scalable deposition technique (spray pyrolysis); **C3**: later versions of the device (PortoCell) had an optimized electrolyte flow‐ path to minimize ionic losses and bubble accumulation; **C1** and **C4**: N/A	2014 [43, 53]
16 000	Mo‐doped BiVO_4_/CoPi (FTO‐coated glass) in series with Si‐HTJ PVs	N/A | 3.00^b^ (@0 V, AM 1.5G, 100 mW cm^−2^)	N/A	1000 h in outdoor	**C1**: multi‐photoelectrode window (100 × 64 cm^2^); **C2‐C4**: N/A	2017 [54]
0.5^b^ | 50	α‐Fe_2_O_3_ (FTO‐coated glass)	≈ 0.94b [13] | ≈0.48b (@1.45 V_RHE_, AM 1.5G, 100 mW cm^−2^)	49	1008 h	**C2**: scalable deposition technique (spray pyrolysis); **C3** and **C4**: CFD‐aided optimization electrolyte feed and flow‐path to minimize ionic losses, bubble accumulation, and concentration gradients (CoolPEC cell); **C1**: N/A	2018 [15, 55]
3.2 | 8 × 3.2	α‐Fe_2_O_3_ (FTO‐coated glass)	0.65 (@1.45 V, AM 1.5G, 100 mW cm^−2^)	≈0	N/A	**C1**: Multi‐photoelectrode window (8 × 3.2 cm^2^); **C2**: scalable deposition technique (spray pyrolysis); **C3** and **C4**: CFD‐aided optimization of electrolyte feed and flow‐path to minimize ionic losses, bubble accumulation, and concentration gradients (CoolPEC cell)	2018 [56]
1 | 40	LaTiO_2_N/ Particles of NiO_x_/CoO_x_ (FTO‐coated glass)	2.29 | 0.56 (@1.23 V_RHE_, AM 1.5G, 100 mW cm^−2^)	76	110 min	**C2**: scalable deposition technique (electrophoretic deposition); **C3**: supporting electrolyte's concentration was enough to minimize ionic‐transport losses; **C1** and **C4**: N/A	2019 [57]
1 | 25 | 225	BiVO_4_/NiFeO_x_ (FTO‐coated glass)	4.40 | 2.10 | 1.30 (@0.6 V_RHE_, AM 1.5G, 100 mW cm^−2^)	52 (25 cm^2^) | 70 (225 cm^2^)	135 h (25 cm^2^) | 1 h every 3 days (225 cm^2^)	**C1**: multi‐photoelectrode window (9 × 25 cm^2^); **C2**: scalable deposition technique (solution‐combustion process); **C3** and **C4**: N/A	2020 [58]
0.24 | 50	W:BiVO4/CoPi (FTO‐coated glass with Ni grid) in series with Si‐HTJ PVs	4.50 | 1.70 (@0 V, AM 1.5G, 100 mW cm^−2^)	62	10 min	**C1**: use of line‐shaped Ni current collectors deposited on top of FTO, protected by the photoabsorber; **C2**: scalable deposition technique (spray pyrolysis and electrodeposition); **C3**: increase of supporting electrolyte's concentration; **C4**: N/A	2020 [22]
<1 | 41.18	BiVO_4_/NiFeOOH (FTO‐coated glass with Au current collector)	2.80 | 2.20 (@0.6 V_RHE_, AM 1.5G, 100 mW cm^−2^)	≈21	≈5 h	**C1**: Use of finger‐shaped Au current collectors, protected by the photoabsorber; **C2**: scalable deposition technique (electrodeposition); **C3‐C4**: N/A	2020 [38]
5.5 | 4 × 8 × 5.5	Worm‐like nanostructured α‐Fe_2_O_3_ (FTO‐coated glass)	≈ 0.75^b^ | ≈ 0.50^b^ (@1.45 V, real sunlight, ≈100 mW cm^−2^)	≈33	48 h	**C1**: Multi‐photoelectrode window (8 × 5.5 cm^2^); **C2**: scalable deposition technique (hydrothermal method); **C3** and**C4**: The module (CoolPEC Module) was built from the side‐by‐side placing of four optimized CoolPEC cells [15]	2020 [59]
0.25 | 50	Cu_2_O/AZO/RuO_x_ (FTO‐coated glass with line‐shaped Cu grid)	5.10 | 3.10 (@0 V_RHE_, AM 1.5G, 100 mW cm^−2^)	40	60 min	**C1**: Use of line‐shaped Ag current collectors embedded on the FTO, protected by the semiconductor; **C2**: scalable deposition technique (electrodeposition); **C3**: use of a device (PortoCell) with an optimized electrolyte flow‐path to minimize ionic losses and bubble accumulation; **C4**: N/A	2021 [39]
1 | 25	TD‐BiVO_4_ ^c^ (FTO‐coated glass) in tandem with Cu_2_ZnSnS_4_ photocathode	6.60^b^ | 0.60 (@0 V)^b^, ^c^	90	7 × 8 h	**C2**: scalable deposition technique (spray pyrolysis and electrodeposition); **C1**, **C3** and **C4**: N/A	2022 [44]
N/A | 25	WO_3_/BiVO_4_/CoPi (FTO‐coated glass with Ni grid)	3.60b | 2.80 (@1.23 V_RHE_, AM 1.5G, 100 mW cm^−2^)	22	80 h	**C1**: Use of line‐shaped Ni current collectors deposited on top of FTO, protected by the photoabsorber; C3: use of an optimized flow‐path reactor; **C2** and **C4**: N/A	2023 [40]
1 | 25	BiVO_4_ (FTO‐coated glass with Ag grid)	0.89 | 0.61 (@1.23 V_RHE_, AM 1.5G, 30 mW cm^−2^)	25	10 h	**C1**: Use of line‐shaped Ag current collectors; **C2**: scalable deposition technique (electrodeposition); **C3** and **C4**: N/A	2023 [60]
N/A | 8000^b^	α‐Fe_2_O_3_ (drilled FTO‐coated glass in assembly with a hydrophobic diffusion layer membrane) in tandem with CuO photocathode	−1.10^b^ [61] | ≈−0.01 (@−0.6 V, real sunlight, ≈100 mW cm^−2^)	99	≈ 6 h	**C2**: scalable deposition techniques (hydrothermal and electro‐reduction methods); **C3**: Optimization of the glass substrate used in the photoanode so as to minimize ionic losses and bubble accumulation; **C1** and **C4**: N/A	2023 [62]
1 | 100	W: BiVO_4_/NiFeOOH (Ti porous transport layer) in series with Si PV	2.30 | 2.10 (@0 V, AM 1.5G, 100 mW cm^−2^)	≈10	6 h	**C1**: use of a metallic substrate; **C2**: scalable deposition technique (SILAR); **C3–C4**: N/A	2023 [7]
0.28 | 49	α‐Fe_2_O_3_ (Micro‐structured FTO‐coated glass with FTO line‐shaped current collectors)	0.63 | 0.63 (@1.45 V_RHE_, 100 mW cm^−2^)	≈0	≈ 1000 h	**C1**: Use of line‐shaped FTO current collectors deposited on top of FTO; **C2**: scalable deposition technique (spray pyrolysis); **C3**: use of a device (PortoCell) with an optimized electrolyte flow‐path to minimize ionic losses and bubble accumulation; **C3** and **C4**: optimization of electrolyte concentration and flow‐rate	**THIS WORK**

^a^ The area considered is the active area of the photoabsorber material (s).

^b^ Estimated or calculated from the reported data.

^c^ Thermoelectric device (TD) was coupled to the PEC cell to enhance the photo‐induced photovoltage. A small‐area PEC system was tested indoors under AM 1.5G, 100 mW cm^−2^ (@0 V), while the large‐area substrates were tested outdoors with natural seawater [44]. N/A—information not available at the source report.

Recent reports have tried to identify and quantify the main sources of performance loss in upscaled PEC‐WS systems [15, 19, 22]. The most relevant sources of inefficiencies seem to include: (1) ohmic losses in the conductive substrate of the photoelectrode; (2) inhomogeneous photoabsorber deposition; (3) ionic losses imposed by the electrolyte conductivity; (4) photoactive area losses related to gas bubble formation/accumulation; and (5) concentration polarization losses at the (photo)electrode–electrolyte interface (including those related to pH gradients). Table 1 displays representative demonstrations of upscaled SCLJ PEC‐WS, with an emphasis given to the strategies adopted to minimize the performance loss observed during upscaling. To better compare and discuss the strategies presented, the aforementioned sources of inefficiencies were grouped into four core challenges:

### 1.1 Ohmic Losses Imposed by the Substrate—Challenge #1 (C1)

The upscaling (by area) of any (photo)electrochemical device leads to increased ohmic losses, as charge carriers must travel longer distances to reach any given interface. The more resistive the materials used in the preparation of the (photo)electrodes and electrical contacts, the higher the toll that ohmic losses will have on the overall performance of the device. PEC‐WS photoelectrodes, especially those of SCLJ systems, are usually prepared on transparent conductive oxides (TCOs) [23]. The use of this class of functional materials is continuously growing for various optoelectronic applications (e.g., flat panel displays, touch screens, and photovoltaics) [24, 25]. Their most appreciated characteristics are high visible light transparency, electrical conductivity, and remarkable chemical, thermal, and mechanical stability. For example, fluorine‐doped tin‐oxide (FTO) is an n‐type metal‐oxide that can exhibit optical transparencies above 80% (400–1000 nm) [24, 26], with a wide energy bandgap (*ca. *3.6 eV), (electro)chemical stability in acidic and alkaline environments [24, 27], thermal stability up to 700 °C (quartz substrate) [25] and sheet resistances (*R*_sheet_) of 7–15 Ω □^−1^ (depending on film‐thickness, fluorine dopant concentration and crystallinity) [28]. Despite the relatively low electrical resistivity, upscaled photoelectrodes with electron‐paths above 5 cm have shown overpotentials above 100 mV, even with current densities below 10 mA cm^−2^ [15, 19]. This non‐neglectable energy loss can be addressed through the use of metallic substrates [7, 29], highly‐conductive intermediate layers, or even by increasing the thickness of the TCO. However, these solutions compromise the optical transmittance of the photoelectrode making them unsuitable for PEC‐PV tandem architectures. Alternative TCOs, like indium tin oxide (ITO) or aluminum‐doped zinc oxide (AZO), offer lower sheet resistances (below 6 Ω □^−1^) [30, 31], but their use is limited by reliance on critical raw materials (e.g., indium) and/or poor chemical and thermal stability [32, 33]. As a result, they are generally incompatible with long‐term, upscaled PEC‐WS operation. A more promising approach, adapted from PV technologies [34] involves incorporating highly‐conductive (typically metallic) busbars across the substrate. These structures serve as current collectors, effectively dividing an upscaled substrate into smaller sections with a reduced electron/charge travel distance. Common current collector materials include Ni, Au, Cu, Ag, and Cr, usually screen‐printed, sprayed, or electrodeposited at the surface of TCO‐coated glasses. A recently‐reported strategy explored embedding metal current collectors beneath the TCO layer, within dedicated channels etched into soda‐lime glass using a photolithography‐assisted process, named embedded TCO (ETCO) [35]. The exceptionally low electrical resistivity of these materials (*ρ*, usually in the range of 10^−6^ Ω cm [36]) considerably reduces the resistance of the substrate, at the expense of some optical transmittance due to the opacity of the collector. The biggest drawback of this solution is the (electro)chemical (ins)stability of these types of collectors when in contact with the corrosive electrolytes commonly found on PEC‐WS systems. Some attempts have been made to mitigate this issue by encapsulating the metal lines with non‐conductive epoxy resins [37] or with the photoabsorber layer itself [22, 38, 39, 40]. However, no reported photoelectrode employing metallic collectors has stayed stable for more than 80 h under continuous operation (Table 1). A recent study from the author's research group [41], proposed using micrometer‐thick FTO lines as current collectors, offering relatively low electrical resistivity (for film thicknesses above 1 µm) while retaining the same thermal and (electro)chemical stability as the FTO‐coated glass itself. Such a solution will be further explored for PEC‐WS applications in this work.

### 1.2 Inhomogeneity of Photoabsorber Layers—Challenge #2 (C2)

Achieving uniform and reproducible deposition of high‐quality large‐area photoelectrodes (including TCO, photoabsorber, catalysts, protection layers, etc.) is a non‐trivial task. As seen in the ongoing industrialization process of third‐generation solar cells, major engineering advances are required in both advanced manufacturing techniques and quality control technologies to accelerate the commercialization of PEC technologies [18, 42]. There is a true technological and performance “gap” between the high‐cost, high‐quality, and time‐consuming techniques that dominate the laboratory scale (i.e., atomic‐layer deposition—ALD, chemical vapour deposition—CVD, molecular‐beam epitaxy, magnetron sputtering, among others) and the relatively rapid, low‐cost but lower‐quality deposition methods that can be easily scaled (i.e., spray‐coating, inkjet‐print, screen‐print, among others). Narrowing this “gap” will become more important in the next few years, as the field shifts toward knowledge that can be easily transferred from “lab to fab.” So far, spray deposition procedures, like spray pyrolysis, have shown the ability to produce uniform metal‐oxide thin films over large areas (up to 100 cm^2^) with good reproducibility [15, 43, 44]. Similarly, (photo)electrodeposition has been widely used for preparing photoabsorber and catalytic layers on substrates as large as 50 cm^2^ [22, 38, 39]. In such processes, incorporating conductive current collectors can significantly improve film uniformity by distributing the applied potential more evenly across the substrate. A recent report explored the idea of using commercially deployable inkjet printing for the deposition of metal‐oxide semiconductors, catalysts, current collectors, and encapsulants on panels as large as 500 cm^2^ [45]. Despite lacking a comprehensive (photo)electrochemical characterization of the prepared materials, this impressive proof‐of‐concept will hopefully quick‐start the development of (semi‐industrial protocols) for the preparation of PEC panels. A more detailed analysis of large area deposition techniques relevant to PEC‐WS might be found elsewhere [8, 46].

### 1.3 Ionic Losses and Gas Bubble Formation/Accumulation—Challenge #3 (C3)

Ohmic losses imposed by low/difficult ionic mobility at the electrolyte (here referred to as “ionic losses”) represent another key challenge during device scale‐up. Such losses are heavily influenced by the electrolyte's ionic conductivity (affected, among other factors, by electrolyte concentration and viscosity), by the membrane characteristics, and by the ionic transport distance/length between (photo)electrodes [18, 47]. Highly alkaline or acidic electrolytes are commonly employed in commercial water‐electrolysis systems, as well as in most PEC‐WS demonstrations, to maximize ionic conductivity (usually 1 M KOH or 1 M H_2_SO_4_, ionic conductivities of *ca. *200 and *ca. *400 mS cm^−1^ at 20–25 °C [15, 48]). While neutral‐pH electrolytes offer an environment less prone to corrosion, they generally require a buffering agent or other supporting electrolytes to achieve acceptable ionic conductivities [19, 22]. Recent reports [15, 19] have shown that overpotentials exceeding 200 mV can be observed in electrodes just a few centimetres apart (<9 cm) operating at current densities <10 mA cm^−2^. This highlights the importance of minimizing ionic transport distances through compact cell designs during scaling. Finally, the adhesion and accumulation of gas bubbles on gas‐evolving (photo)electrodes is a common issue in large‐area systems. This phenomenon can lead to losses of (photo)active area and cause unwanted light reflection and scattering, ultimately degrading device performance [47, 49]. In this regard, multi‐phase computational fluid dynamics (CFD) simulations offer valuable insights to better design devices whose architecture and operational parameters (like flow rate and internal pressure) can help maximize (gas) product collection and separation [15, 21].

### 1.4 Losses From Concentration Polarization and pH Gradients—Challenge #4 (C4)

When an electrochemical reaction is fast enough to lower the concentration of reactants at the surface of the electrode below that of the bulk solution, a concentration gradient is established. This gradient induces an overpotential known as concentration polarization, which can be described by the Butler–Volmer equation [50]. In systems where redox reactions involve H^+^ and OH^−^ ions, the previously mentioned concentration gradient will also translate into a pH gradient near the (photo)electrode surface. This effect is particularly pronounced in neutral‐pH electrolytes, where the concentration of these ionic species is inherently low. The resulting pH gradient can negatively impact reaction kinetics by increasing the thermodynamic equilibrium potential of the system, leading to the so‐called Nernstian losses/overpotentials [19, 50]. When concentration and/or pH gradients are observed in a system, one of the main factors influencing the rate of the ongoing reaction is mass transport (the rate at which the reactants and products are transported to and from the electrode) [51]. As such, strategies that help maximizing mass transport are a common way to reduce the previously‐mentioned overpotentials. Forced electrolyte stirring and recirculation have been shown to induce convective mass transport [19, 22, 52]. This effect can be further amplified by designing optimized electrolyte flow channels/paths that promote turbulence and/or maximize bubble formation/circulation (bubble‐induced convection) [21].

This work aims to develop an optimized 49 cm^2^ PEC‐WS device capable of addressing the most severe upscaling challenges observed in this technology. For this end, a lift‐off lithography‐assisted spray pyrolysis method was developed to produce large‐area FTO‐coated glass substrates with improved uniformity, conductivity, surface area, and stability. Strategies including substrate microstructuring and the integration of micrometre‐thick FTO current collectors were implemented to mitigate performance‐limiting factors such as ohmic resistance and low surface area. Furthermore, device‐level engineering was applied to address ionic transport limitations, gas bubble accumulation, and interfacial concentration and pH gradients.

## 2 Results and Discussion

**FIGURE 1:**
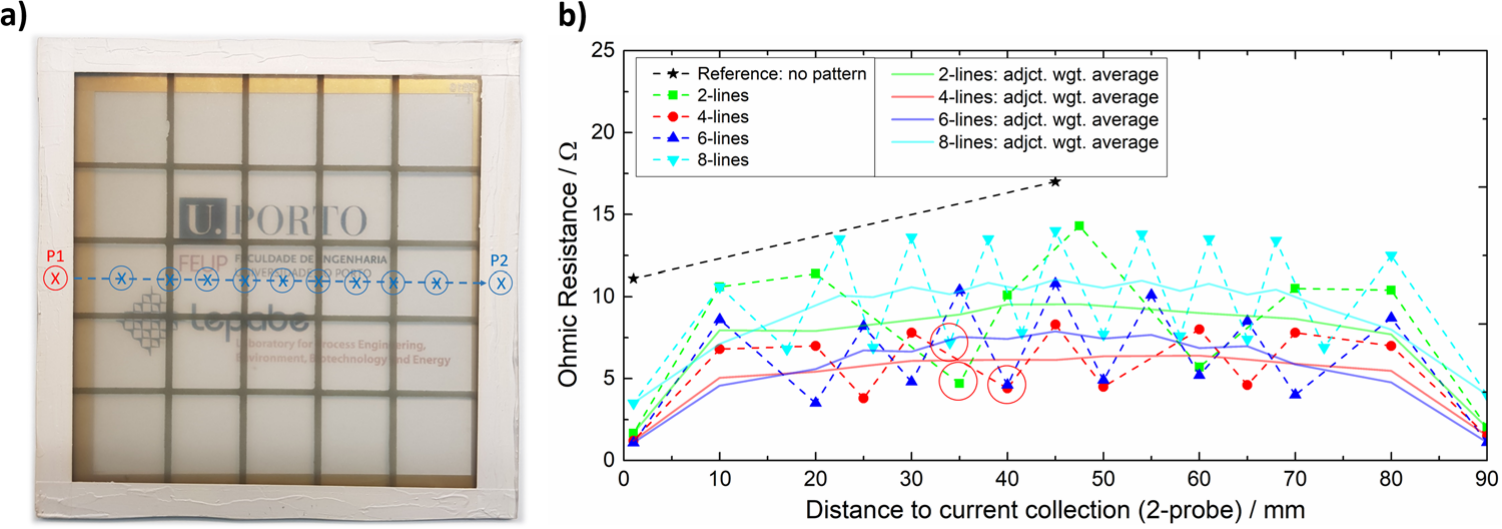
(a) Schematic illustration for the two‐point probe (2PP) resistance measurements: static probe (P1) on the frame, moving probe (P2) across the substrate (here exemplified for sample “4‐lines”); (b) experimental results for the substrates under study. Data shown as both raw (markers) and adjacent weighted averages (solid lines). Red circles highlight measurements directly over collector lines, for similar probe distances.

**FIGURE 2:**
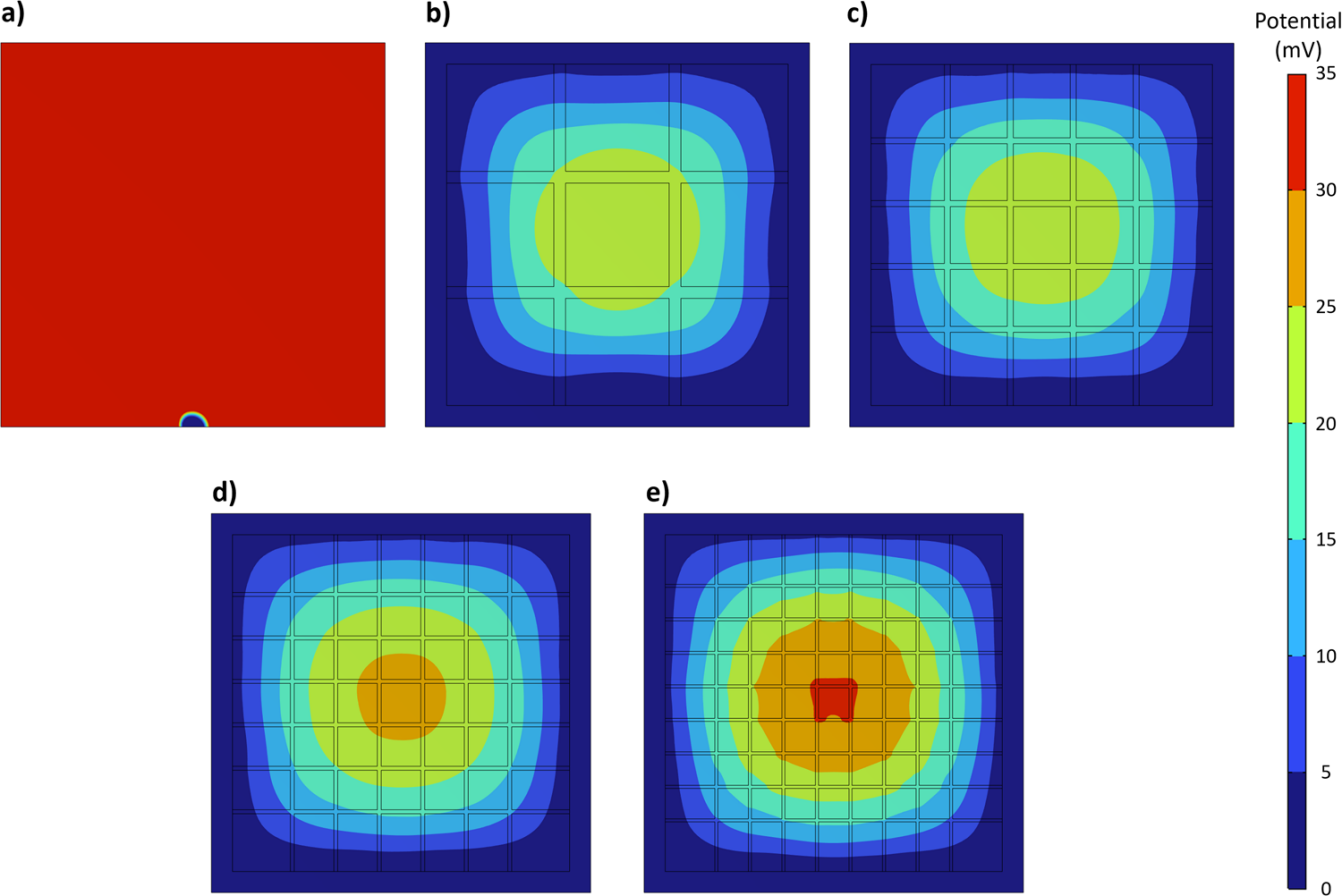
Simulated overpotential gradient contours calculated using the proposed conductivity model and the 9 × 9 cm^2^ substrates under study: (a) commercial FTO‐coated glass with no current collectors; and substrates with (b) 2; (c) 4; (d) 6; and (e) 8 horizontal/vertical FTO current collectors.

Figure 1 compares the characteristic current density *vs* potential curves (*J*–*E*) curves for small‐area (*ca. *0.28 cm^2^) and large‐area (*ca. *49 cm^2^) thin‐film α‐Fe_2_O_3_ photoelectrodes (PEs), both prepared and characterized under similar experimental conditions. As shown, the large‐area photoelectrode exhibits a noticeable performance loss of *ca. *45% in the photogenerated current density at 1.45 V_RHE_ compared to its small‐area counterpart. Such a phenomenon has been widely observed in PEC‐WS literature (Table 1), and is mostly attributed to a combination of manufacturing and transport‐related challenges as outlined in the Introduction. One of the main challenges in upscaling PEC devices is the increase in ohmic losses imposed by conventional TCO substrates (Challenge #1). To better understand this limitation, an electronic conductivity simulator (COMSOL Multiphysics) was used to model the potential distribution across a 9 × 9 cm^2^ FTO‐coated substrate with a resistivity of *ca. *4.5 × 10^−4^ Ω. Figure 2a shows the resulting overpotential gradient when a current source (matrix of points—Figure S1a) in the substrate generates a current of 1 mA. A potential drop on the order of tens of mV emerges near the substrate edges, a serious limitation that would be even more aggravated if higher currents were considered. High conductivity/low resistivity current collectors are the most common approach to mitigate this challenge. However, conventional metal‐based materials used in this context are prone to (electro)chemical corrosion and/or adhesion‐related problems [25].

### 2.1 Thick‐Layered FTO as a Current Collector for PEC Applications

A recent report from the authors of this work explored the idea of using micrometre‐thick, highly‐conductive FTO current collectors to enhance the lateral conductivity of TCO‐based substrates [41]. Such an approach takes advantage of some of the main merits of this material, namely: (1) the relatively low electrical resistivity of micrometre‐thick films (as low as 10^−5^ Ω cm [63]); (2) proven stability in aqueous solutions with pH ranging from 0 to 14 [24]; and (3) compatibility with scalable, low‐cost, low‐waste deposition techniques such as spray pyrolysis. Preliminary experiments conducted on SCLJ devices explored two‐dimensional (2D) grid‐collector geometries/patterns based on polygons that tessellate: triangles, squares, and hexagons. Both simulated and experimental data showed that the geometry of the collectors plays a pivotal role in how and with what efficiency current extraction takes place. For a fixed collector coverage, square‐shaped patterns seemed to guarantee shorter and most direct charge‐extraction pathways and, therefore, the smallest potential drops.

This work further explores the concept of FTO line current collectors, organized in squared‐shaped patterns. The previously‐mentioned conductivity simulator (explained in detail in Section 4) was used to optimize this design, following a set of strict boundary conditions: all current collectors (1) were deposited on an initial FTO‐coated glass substrate with a resistivity of *ca. *4.5 × 10^−4^ Ω cm [41]; (2) had *ca. *5 µm of height (resistivity of *ca. *3.2 × 10^−4^ Ω cm, experimentally measured in [41]); (3) covered only *ca. *15% of the substrate area; and (4) were connected to a 0.5 cm‐wide FTO perimeter frame coated with silver paste for improved electrical contacts [64]. Following a stepwise optimization process, simulations were conducted with varying numbers of horizontal and vertical current collector lines.

Figure 2b–e presents the simulated (over)potential contours of substrates considering 2, 4, 6, and 8 vertical/horizontal lines of FTO current collectors. For all the designs, incorporating the current collectors significantly reduces the overpotentials imposed by the TCO when compared with the collector‐less reference (Figure 2a). Notably, increasing from 2 to 4 vertical/horizontal lines yields substantial gains in charge extraction at both the perimeter and center of the sample. However, for simulations with 5 and 7 lines (Figure S2), a higher density of lines does not translate into further improvements in charge collection. To better understand this phenomenon, substrates considering 2, 4, 6, and 8 vertical/horizontal lines of FTO were subjected to two‐point probe (2PP) resistance measurements. During these tests, one probe was fixed at the edge/frame, while the second probe moved along the length of the substrate, measuring the electrical resistance as illustrated in Figure 1a. As expected, the resistance gradually increases with probe distance, reaching a maximum value at the center of the sample—Figure 1b. Each time the moving probe is placed over a line/current collector, the resistance falls drastically. To facilitate the comparison among the different samples, an adjacent weighted average method was applied to obtain a smoother data plot. The results (Figure 1b solid lines) show that using current collectors drastically reduces the resistance felt across the substrate. The best‐performing samples record less than 7.5 Ω at the center of the sample, *ca. *45% of the resistance for a collector‐less reference. As observed in the simulations, the configuration with 4 vertical/horizontal lines leads to an overall less resistive substrate (hereafter referred to as “4‐lines”), especially at the center of the sample. The experimental data also confirms that the “8‐lines” sample is the least effective design. This result might be explained by the very narrow width (*ca. *0.5 mm) of the lines employed in this design to comply with the boundary condition of the current collecting lines, covering only *ca. *15% of the total substrate area. Such thin lines are harder to manufacture without defects and are less efficient in electron transport compared to wider alternatives. This conclusion seems to be supported by the fact that the 2PP resistance measurements taken over the lines/collectors on “8‐lines” showed the highest resistance among all samples (highlighted with red circles in Figure 1b).

**FIGURE 3:**
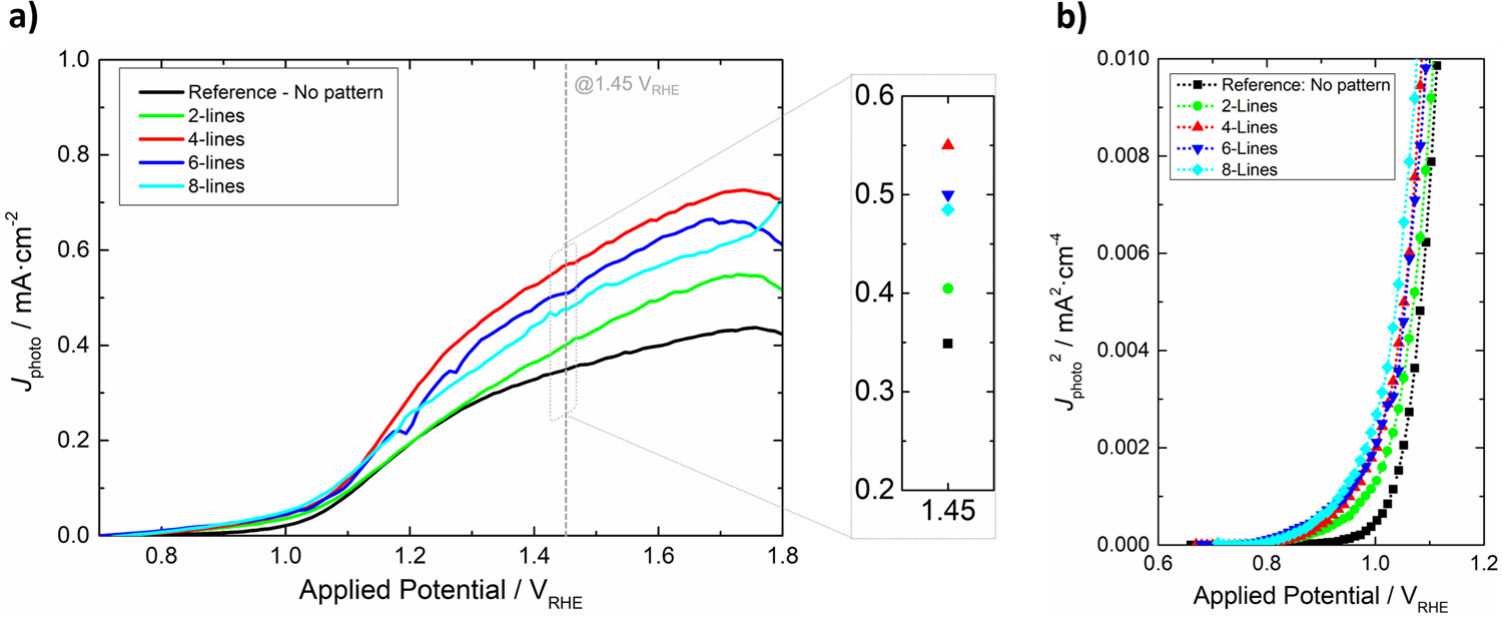
(a) Light‐induced photocurrent density (*J*_photo_) as a function of applied potential for the bare α‐Fe_2_O_3_ photoelectrodes prepared on different FTO‐coated glass substrates with varying FTO current collector configurations; (b) *J*_photo_^2^ versus *E* plots used to estimate the photocurrent onset potential, determined by the horizontal intercept of the extrapolated linear region (when *J*_photo_^2^ = 0).

Thin‐film bare α‐Fe_2_O_3_ photoelectrodes were prepared using the proposed substrates (photoactive area of 7 × 7 cm^2^) and characterized by *J*–*E* measurements. The light‐induced photocurrent *J*_photo_ could be estimated from the raw *J*–*E* curves (*J*_light/dark_) using Equation S1, and plotted as a function of the applied potential—Figure 3a. All samples exhibit a *J*_photo_ onset potential between 1.01 and 1.06 V_RHE_ ‐ Figure 3b. This narrow potential range (relative standard deviation from the mean of <2%) is in line with previous reports, being determined by kinetic and thermodynamic factors at play at the SCLJ [65]. The proposed FTO current collectors, deposited on the glass substrates prior to α‐Fe_2_O_3_ deposition, are not expected to influence these factors at the SCLJ. Nevertheless, for applied potentials above *ca. *1.30 V_RHE_, samples incorporating current collectors exhibit significantly higher photocurrents due to enhanced charge‐extraction efficiency ‐ Figure 3a. Notably, the photoelectrode prepared using the “4‐lines” substrate produced a *J*_photo_ of *ca. *0.55 mA cm^−2^ at 1.45 V_RHE_, 57% above the value observed for the collector‐less reference (Table 2). The fill factor (FF) is also affected by the collector's design, generally increasing with the number of lines. The highest FF is observed for the “8‐lines” sample; however, the comparatively high resistance of its substrate hinders the generation of photocurrent at potentials >1.15 V_RHE_. As a result, the *J*_photo_ of “8‐lines” at the reference potential of 1.45 V_RHE_ lags behind the one observed for other samples. The superior performance “4‐lines” seems to correlate well with the fact that this design shows the lowest simulated (over)potentials (Figure 2) and experimental resistances (Figure 1), both at the centre and perimeter of the upscaled substrate.

**TABLE 2:** Summary of photoelectrochemical performance metrics for bare α‐Fe_2_O_3_ photoelectrodes prepared on different FTO‐coated glass substrates with varying FTO current collector configurations.

Sample	*J*_photo_ onset (V_RHE_)	*J*_photo_ _@1.45 VRHE_ (mA cm^−2^)	FF
Ref.	1.06	0.35	0.39
2‐Lines	1.04	0.40	0.30
4‐Lines	1.03	0.55	0.34
6‐Lines	1.02	0.50	0.32
8‐Lines	1.01	0.49	0.41

### 2.2 Micro‐Structuring FTO‐Coated Glass Substrates

**FIGURE 4:**
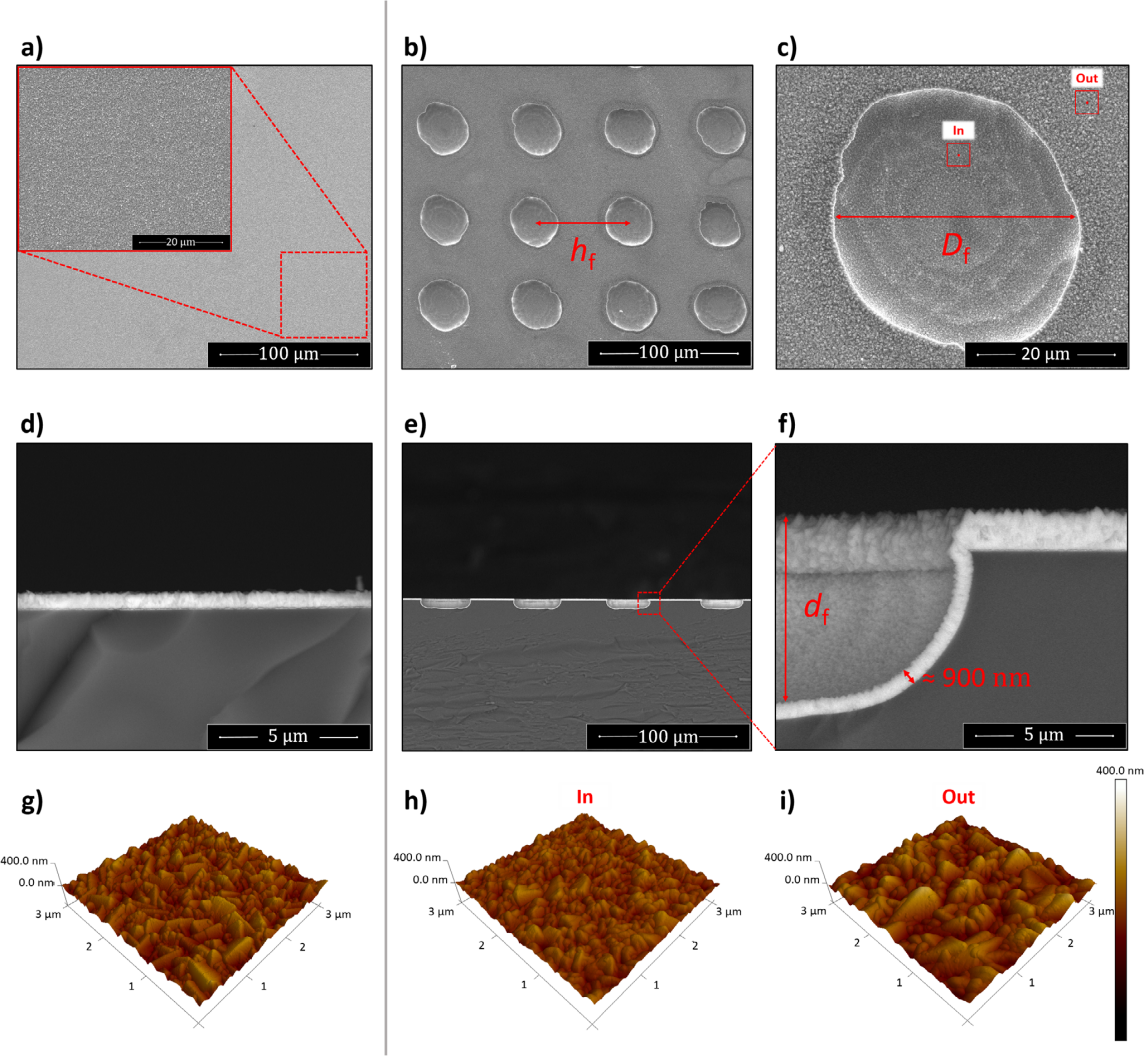
Top‐view and cross‐section SEM imagery of flat (a,d) and micro‐structured (“A‐60‐30”: (b,c,e,f) FTO‐coated glass substrates. Three‐dimensional detailed surface morphology obtained by AFM for a 3 × 3 µm^2^ region of the same flat (g) and micro‐structured FTO‐coated glass substrates, the latter analysed inside (h) and outside (i) of a micro‐scale groove.

Despite the proven effectiveness of the proposed line‐shaped FTO current collectors, the photocurrent density at 1.45 V_RHE_ of the best‐performing design was experimentally found to be only *ca. *87% of that measured for a smaller photoelectrode (area of *ca. *0.28 cm^2^). To decrease further the performance gap between the small‐ and the large‐area photoelectrodes, this work explores an additional engineering strategy—the surface texturization of the FTO‐coated glass substrate. Commercial FTO (TEC‐7) has a characteristic 2D topography, with a very mild roughness from the nanometre‐scale surface grains [24]—Figure 4a,f. The 3D‐structuring of this layer has long been considered a promising strategy to improve the PEC performance of photoelectrodes prepared in such substrates. A larger FTO surface area increases the number of active sites, increasing the rate of the redox reactions under study; in addition, it provides more interfacial area for semiconductor heterojunction formation and catalyst/dopant loading [23]. 3D TCO layers are often produced through template‐assisted procedures or through nano/micro‐imprinting lithography [66]. Template‐assisted approaches involve depositing the TCO onto a sacrificial template, such as polystyrene (PS) [67], SiO_2_ nanospheres, or even porous anodic aluminum oxide (AAO) [23, 68], which is subsequently removed by chemical or physical methods. In the latter technique, a stamp (usually Si‐based) is used to imprint a polymer mold with the desired (usually nano‐scale) pattern. This mold is either used as a template to directly grow a TCO on top [69, 70] or to selectively etch an already‐existing TCO layer with the reverse original pattern [71].

**FIGURE 5:**
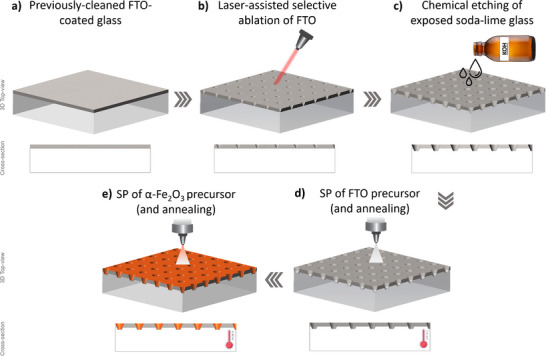
Schematic illustration of the experimental procedure used in the preparation of micro‐structured FTO‐coated glass substrates.

In this work, we propose a hybrid approach that combines elements of both laser‐ablation lithography and template‐assisted etching to fabricate micro‐structured FTO‐coated glass substrates – Figure 5. First, a laser beam is used to selectively ablate regions from the FTO layer on a commercial FTO‐coated glass, originating dot‐shaped cavities in a square‐shaped array. Afterward, the remaining FTO sections of the substrate are used as a template in a chemical etching treatment that further expands the size and depth of the cavities. In the final step, the entire substrate, both etched and non‐etched regions, is coated with a *ca. *900 nm‐thick FTO layer via spray pyrolysis—Figure 4f. The formed cavities/features (Figure 4b–e) have a circular groove/cavity‐like shape, with an external diameter (*D*_f_) in the range of 30–60 µm, depths (*d*_f_) of 10–30 µm, and relative distance/pitch (*h*_f_) of 0–30 µm. The mentioned geometrical characteristics were tuned by varying two key experimental parameters: (i) the size of the laser‐ablated square region (keeping laser power, scanning speed, and pulse count constant); and (ii) the chemical etching duration (keeping constant solution composition and temperature conditions). Following a stepwise optimization process, a range of micro‐structured FTO‐coated substrates (initially prepared on 1.2 × 3 cm^2^ glass) were fabricated and labeled based on their specific fabrication parameters, as summarized in Table 3.

**TABLE 3:** Key experimental conditions used in the preparation of micro‐structured FTO‐coated glass substrates and sample identification (ID).

Laser‐patterned area (mm^2^)	Chemical etching time (s)	Sample ID
60 × 60	30	A‐60‐30
	60	A‐60‐60
	90	A‐60‐90
45 × 45	30	A‐45‐30
	60	A‐45‐60
	90	A‐45‐90
30 × 30	30	A‐30‐30
	60	A‐30‐60

**FIGURE 6:**
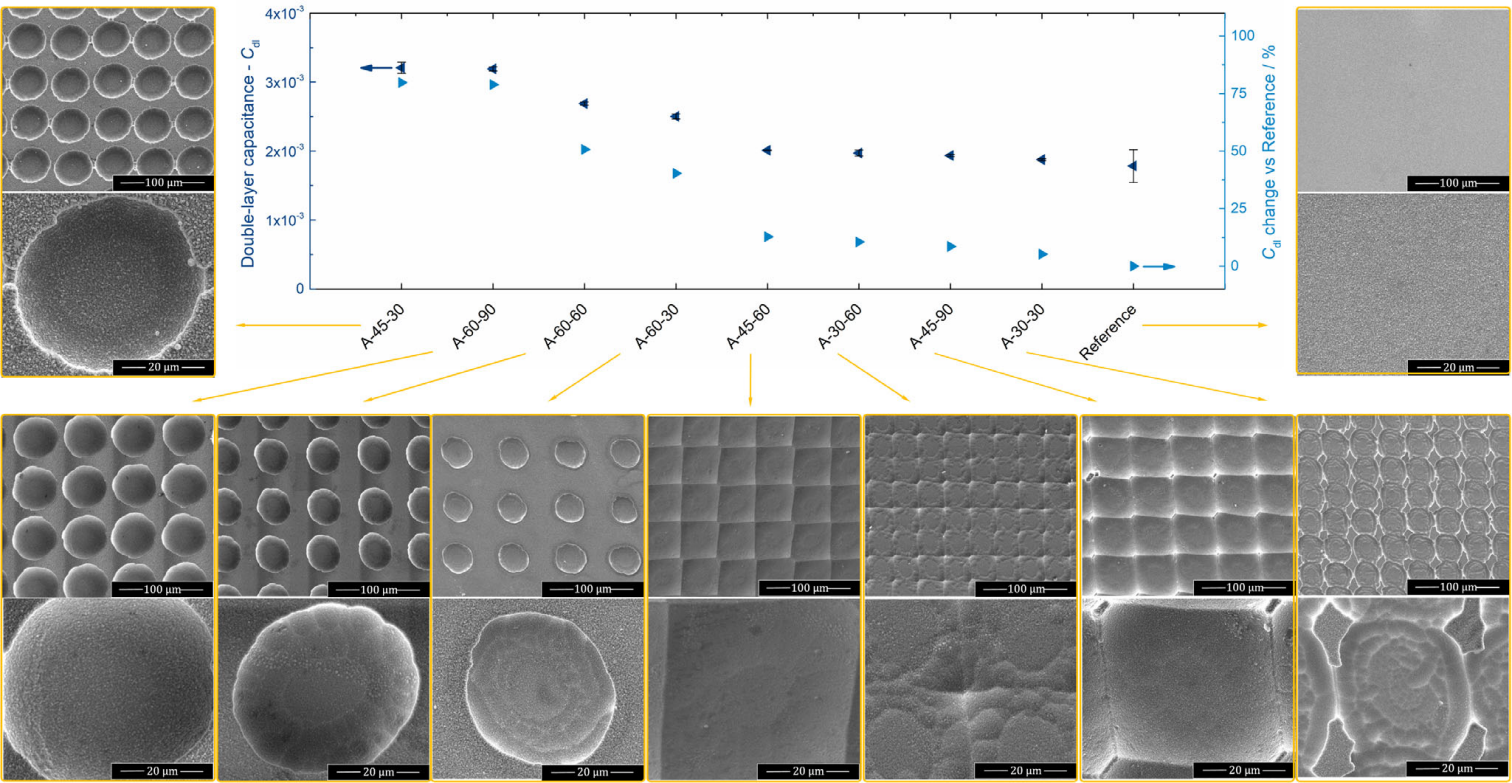
Top‐view high‐resolution SEM imagery (magnification of 1000× and 5000×) and double‐layer capacitance (*C*_dl_) estimated by EIS for the multiple design patterns of micro‐structured FTO‐coated glass substrates. Samples are displayed in descending order of *C*_dl_.

Electrochemical impedance spectroscopy (EIS) was used to estimate the double‐layer capacitance (*C*_dl_) at the open‐circuit potential (*E*_OCP_) of the proposed substrates. This important parameter reflects the formation of the electrical double layer at the electrode–electrolyte interface, which is made of electrons on the electrode side and ions in the electrolyte [72]. The charge stored is a function of, among other factors, the permittivity of the materials involved (*ε*) and the electrochemically accessible surface area. Assuming that the only difference among the micro‐structured FTO substrates is their surface area, *C*_dl_ provides a quantitative metric to estimate the relative increase in surface area due to the introduced micro‐scale features. Figure 6 shows the *C*_dl_ measured for all substrates under study, sorted in descending order. As can be observed, all samples subjected to the micro‐structuring procedure showed a higher *C*_dl_ than a (comparatively) flat commercial FTO‐coated glass reference. For the majority of samples, there seems to be a direct correlation between the surface area increment and the size and density of features. This is true while the groove‐like features are still well‐defined and individualized. In contrast, samples that are the result of a coalescence of features, like those where the initial laser‐ablated grooves had the lowest pitch (*h*_f_ of *ca. *45 µm—“A‐30” series) and/or those that suffered a long chemical etching (“A‐45‐60” and “A‐45‐90”, Table 3), deviate from this trend since their surface morphology is quite flat. Nevertheless, certain designs, such as “A‐45‐30” and “A‐60‐90” series, show that the proposed micro‐structuring procedure is able to increase the surface area of FTO‐coated glass substrates by >70%.

In addition to offering increased surface area, an ideal micro‐structured substrate should preserve key properties, namely, high optical transparency and low electrical resistance. To evaluate these aspects, the optical transmittance of the proposed substrates was measured via UV–vis–NIR spectrophotometry (Figure S8), while a pseudo sheet resistance ($R_{\mathrm{sheet}}^{\prime }$) was estimated using the van der Pauw method. The term “pseudo sheet resistance” is applied since the proposed micro‐structured films do not fully meet the van der Pauw method's requirement of a thin film with homogeneous thickness. As such extracted values should be interpreted only for relative comparison across samples measured under identical conditions.

Figure S9 compares the transmittance at *λ* = 400 nm (wavelength at which α‐Fe_2_O_3_ has maximum light absorption) and the $R_{\mathrm{sheet}}^{\prime }$ of the proposed micro‐structured substrates with a reference flat FTO‐coated glass. In general, samples with larger feature size (both in diameter and depth) and/or density (lower pitch) exhibited higher optical transmittance and high electrical resistance. This phenomenon can be attributed to a thinner average FTO layer on said samples, due to the removal of more native FTO in the preparation procedure. Conversely, samples with smaller or more sparsely spaced features had more regions of their surface where the FTO film was thicker (*ca. *500 nm of native FTO + *ca. *900 nm of FTO sprayed at the end of the proposed micro‐structuring procedure); as a result, such samples demonstrate a lower resistance but also reduced light transmission. Despite these variations, all substrates under study maintained an acceptable balance: light transmittance at 400 nm never dropped below 40% (compared to *ca. *70% for commercial TEC‐7 FTO‐coated glass), and $R_{\mathrm{sheet}}^{\prime }$ values remained below 10 Ω □^−1^.

**FIGURE 7:**
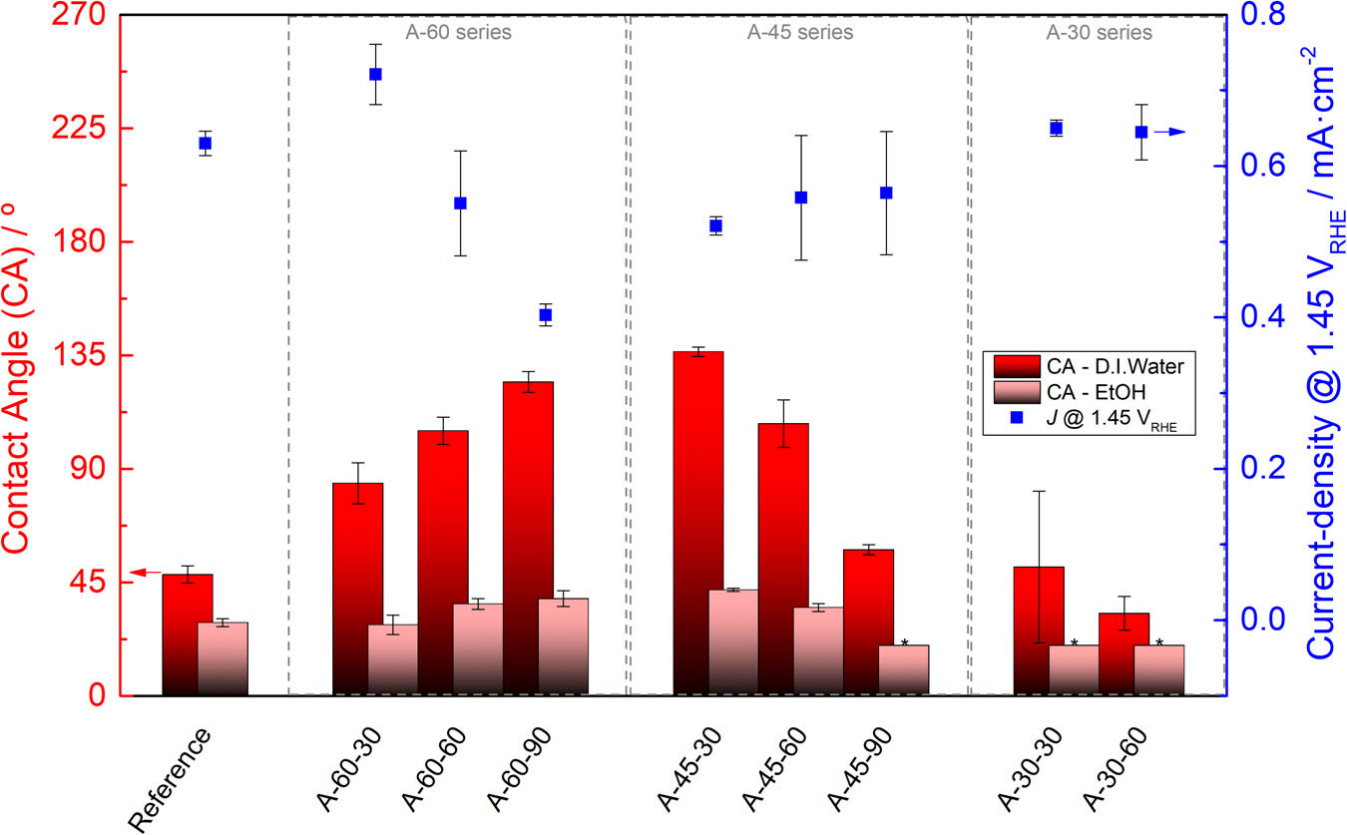
Contact angle (CA) measurements with distilled water and ethanol (in red) and photo‐generated current density (*J*) at 1.45 *V*_RHE_ (in blue) for α‐Fe_2_O_3_ photoelectrodes prepared from the micro‐structured FTO‐coated glass substrates under study. * Note: The ethanol (EtOH) contact angles for samples A‐45‐90, A‐30‐30, and A‐30‐60 were very low (<20°), making it difficult to obtain representative and consistent measurements across replicates.

Surface wettability was also evaluated using contact angle measurements with distilled water (H_2_O) or ethanol (EtOH) droplets ‐ Figure 7 (red). The results revealed that surface roughness significantly influences the interaction between the substrates and liquid. Most of the micro‐structured patterns exhibit a higher contact angle (lower wettability) to H_2_O and EtOH than the flat reference. The higher the size and density of features, the higher the contact angle; the same trend was observed with surface area. The highest contact angles to H_2_O (well above 90°) were observed for samples “A‐60‐90” and “A‐45‐30”, the ones predicted to have the highest surface area. Such a phenomenon was expected since top‐down micro‐scale texturization treatments have been used to decrease the wettability of surfaces made of various materials (e.g., metallic, semiconductor, and polymeric) [73, 74]. Low wettability is an undesirable feature for substrates used in PEC‐WS applications, as will be discussed later.

To determine whether any of the proposed micro‐structuring patterns could successfully leverage surface area enhancement without being critically compromised by poor wettability, a thin‐film of α‐Fe_2_O_3_ was spray‐coated onto the proposed substrates (1.2 × 3 cm^2^) and characterized for water oxidation via *J‐*‐*E* measurements. Figure 7 (blue) highlights the photocurrent density measured at 1.45 V_RHE_ for all samples under study. Despite the higher surface area (Figure 6), most of the proposed micro‐structured photoelectrodes generate lower photocurrent densities than the flat reference. Interestingly, when these photocurrent values are compared with the corresponding contact angles (Figure 7, red), there seems to be a clear negative/reverse correlation. For substrates that retained well‐defined grooves, a higher number and density of these features lead to a decrease in photocurrent, which is the opposite of what is observed for H_2_O and EtOH contact angles. When the coalescence of grooves is promoted (like on the “A‐30” sample series), this trend is not observed since the surface becomes increasingly flat. These results suggest that gains in surface area via micro‐structuring often come at the cost of wettability, which may hinder PEC performance, although other micro‑structuring‑induced effects cannot be excluded.

Poor wettability, associated with hydrophobic surfaces, is known to prevent gas bubble detachment, thereby penalizing the reaction rate [75, 76]. Similarly, a low affinity to EtOH negatively affects the deposition of semiconductor, dopant, and/or catalyst layers that use this compound in liquid precursor solutions. Substrate roughness has shown to affect the solution spreading and evaporation processes in wet chemistry depositions, thus disturbing the crystallization energy barrier [77]. To assess this hypothesis, energy‐dispersive X‐ray spectrometry (EDS) measurements were performed on regions inside and outside the grooves of a representative “A‐60‐90” sample (one of the highest predicted surface areas but lowest wettability). As shown in Figure S10, the elemental composition of these two substrate regions is slightly different. The region inside the grooves has a lower amount of iron (Fe) and oxygen (O), seemingly confirming the detrimental effect that low wettability has on the α‐Fe_2_O_3_ crystalization process. Despite this drawback, some micro‐structured substrates outperformed the flat reference in terms of photocurrent density. A clear example is the “A‐60‐30” sample series that delivered an average photocurrent density of *ca. *0.72 mA cm^−2^ at 1.45 V_RHE_. Such a result is *ca. *18% superior to the reference, proving that a good compromise can be achieved between surface area increase and wettability loss.

### 2.3 Synergetic Effect of FTO Current Collectors and Micro‐Structuring on 49 cm^2^ Substrates

The enhanced conductivity offered by the proposed FTO line‐shaped current collectors (here labeled as “CC”) and the increased surface area achieved through the optimized micro‐structuring pattern (“MSP”) were combined in a single 49 cm^2^ substrate. After depositing a thin film of α‐Fe_2_O_3_, the resulting photoelectrode (designated “A‐CC‐MSP”) was evaluated for PEC water‐oxidation through *J*–*E* measurements in a three‐electrode setup (experimental details provided in Figure S4). To isolate the contribution of each individual substrate engineering solution, the performance of the optimized photoelectrode was benchmarked against three other photoelectrodes of equal dimensions prepared from: (1) a flat commercial FTO‐coated glass substrate (TEC‐7, labeled as “Reference”); (2) a micro‐structured FTO‐coated glass substrate employing the “A‐60‐30” pattern design (labeled as “A‐MSP”); and (3) a flat FTO‐coated glass substrate integrating line‐shaped FTO current collectors (labeled as “A‐CC”, previously referred to as “4‐lines” in Section 2.1).

**FIGURE 8:**
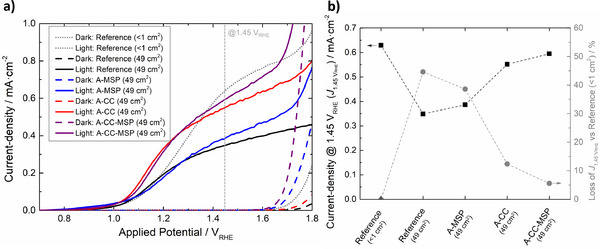
(a) Dark and light (1‐sun) *J*–*E* curves for small (<1 cm^2^) and large‐area (49 cm^2^) thin‐film α‐Fe_2_O_3_ photoelectrodes prepared on the different FTO‐coated glass substrates under study; (b) photocurrent density (at 1.45 *V*_RHE_) measured for the same samples (in black) and their relative value when compared with Reference (<1 cm^2^, in grey).

As displayed in Figure 8a, the micro‐structured photoelectrode A‐MSP outperformed the Reference for applied potentials above *ca. *1.30 V_RHE_. At 1.45 V_RHE_, it registered a photocurrent density of *ca. *0.39 mA cm^−2^, representing a 12% increase over the Reference. As expected, further improvements were observed when micro‐structuring was combined with the FTO current collectors. The A‐CC‐MSP photoelectrode displays a photocurrent density of *ca. *0.60 mA cm^−2^ at 1.45 V_RHE_, outperforming the Reference by *ca. *71% and by *ca. *10% the photoelectrode considering just the current collectors (“A‐CC”).

Until this moment, all substrate engineering efforts were capable of delivering a 49 cm^2^ thin‐film α‐Fe_2_O_3_ photoelectrode that displays *ca* 94% of the photocurrent density observed at 1.45 V_RHE_ for a small‐scale counter‐part (0.28 cm^2^) made from a commercial flat FTO‐coated glass—Figure 8b. Despite being an encouraging result, the high ohmic losses and low surface area of the TCO substrates are not the only upscaling challenges of PEC‐WS. Large‐area systems have their performance also hindered by ionic losses imposed by low electrolyte conductivity, losses related to bubble formation/accumulation, and losses related to concentration polarization and pH gradients. To address these additional limitations, the PEC device used for all large‐scale characterizations (PortoCell) was specifically designed to minimize the distance between cell components and to guarantee an effective electrolyte flow that reduces bubble accumulation [43]. Furthermore, to reduce ionic resistance, concentration polarization, and pH gradient losses, both electrolyte concentration and flow‐rate were optimized, as explained in Section S3 (Figures S5 and S6).

**FIGURE 9:**
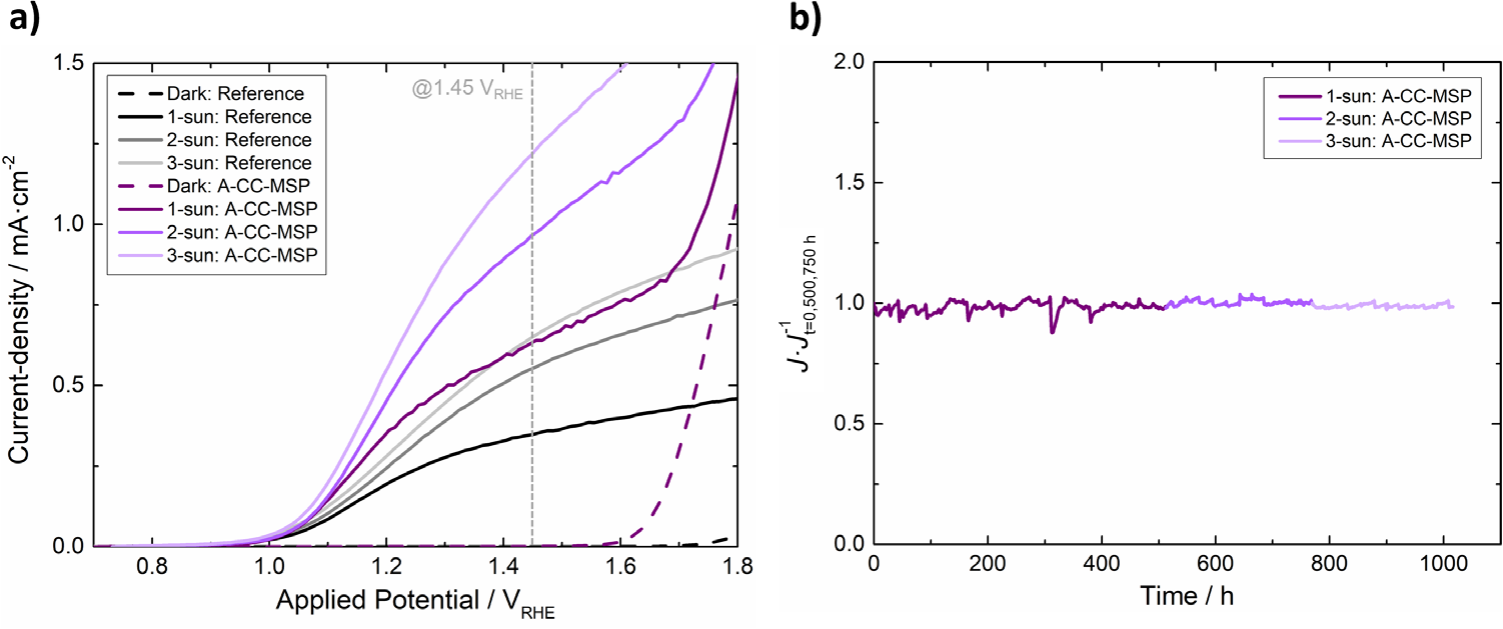
(a) *J–E* curves of Reference and A‐CC‐MSP thin‐film α‐Fe_2_O_3_ photoelectrodes measured under different simulated sunlight intensities (100, 200, and 300 mW cm^−2^, labeled as 1‐, 2‐, and 3‐sun, respectively). The reference sample was tested in 1 M KOH without electrolyte flow, while the A‐CC‐MSP sample was tested in 4 M KOH with a flow rate of 50 mL min^−1^. (b) Normalized photocurrent density history of the A‐CC‐MSP photoelectrode during a 1000 h chronoamperometry test (at 1.45 V_RHE_), conducted under 1‐sun simulated sunlight for the first 500 h, followed by 2‐sun for *ca* 250 h and 3‐sun for the final *ca* 250 h. To facilitate data interpretation, the current density values were normalized according to the current measured at the beginning of each stage.

**FIGURE 10:**
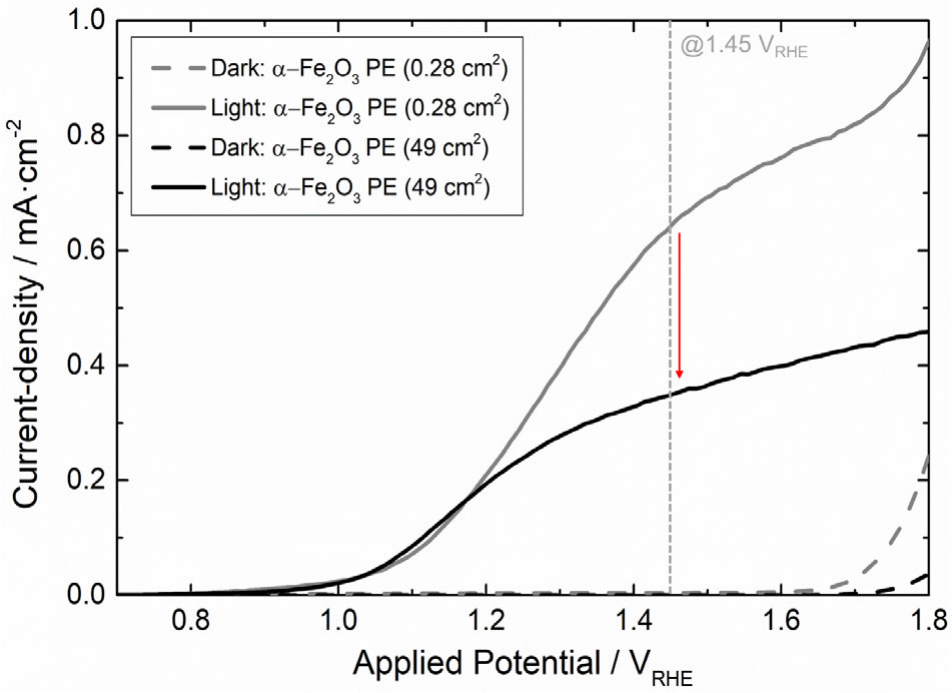
Dark and light (1‐sun) *J–E* curves of thin‐film α‐Fe_2_O_3_ photoelectrodes with small (<1 cm^2^) and large (49 cm^2^) active areas, deposited on commercial FTO‐coated glass substrates.

Figure 9a shows the *J–E* curve of the A‐CC‐MSP photoelectrode, measured under optimized operating conditions: 4 M KOH electrolyte and a flow rate of 50 mL min^−1^. Under these conditions, a photocurrent density of *ca* 0.63 mA cm^−2^ is observed at 1.45 V_RHE_, effectively matching the performance of the small‐area reference photoelectrode (Figure 10, in grey, measured in a stagnant 1 M KOH electrolyte). This result validates the effectiveness of all engineering solutions here employed to tackle the upscaling challenges of PEC‐WS systems. It is reasonable to expect that these improvements would be even more significant for a system employing a photoelectrode built from more efficient photoabsorber layers. In this scenario, the detrimental effect of all charge and mass transport limitations would be even more evident due to higher operational photocurrent densities. To simulate this scenario, the optimized PEC‐WS device equipped with the A‐CC‐MSP photoelectrode was tested under different simulated sunlight intensities: 1‐, 2‐, and 3‐sun. For comparison, a bare α‐Fe_2_O_3_ photoelectrode on a standard substrate (“Reference”) was also tested, but in the absence of electrolyte flow and with a 1 M KOH solution as electrolyte. The resulting *J–E* curves (Figure 9a) confirm a more accentuated photocurrent increase with light intensity for the optimized system, reaching *ca. *0.86 mA cm^−2^ at 2‐sun and *ca* 1.10 mA cm^−2^ at 3‐sun (1.45 V_RHE_).

In the final part of this study, the long‐term stability of the optimized system was evaluated in a 1000 h long chronoamperometry. During this test, the photocurrent was continuously measured at 1.45 V_RHE_, the electrolyte had a concentration of 4 M KOH and a flow rate of 50 mL min^−1^. The photoelectrode was exposed to 1‐sun for the first 500 h, followed by 2‐sun from 500 to 750 h, and 3‐sun during the final 250 h. As shown in Figure 9b, the normalized photogenerated current remained remarkably stable throughout the entire 1000‐h test. An SEM analysis of fresh and aged A‐CC‐MSP samples revealed no significant differences in the surface topography (Figure S11a,b). Cross‐section imagery (Figure S11c,d) confirmed that the 5–6 µm thick, *ca. *1.3 mm wide line‐shaped FTO current collectors have not delaminated or detached from the surface. Similarly, the micro‐grooved regions retained their shape, depth, and FTO coverage. XPS spectra collected for both samples exhibit the peaks related to Fe^3+^ and Fe─O bonds in Fe_2_O_3_ (Figure S12) [78, 79]. A peak fit of the wide XPS spectrum (Figure S11e) confirms that the surface composition of both samples is very similar, showing that a slight degradation of the semiconductor could have occurred (*ca. *20% decrease of the Fe/O surface ratio ‐ Table S2). To further evaluate material stability, electrolyte samples collected throughout the experiment were analysed by ICP‐OES to determine the concentration of dissolved Fe and Sn coming from the photoelectrode. As shown in Figure S13, the concentration of both metals gradually increased throughout the test, reaching concentrations of *ca. *743 µg l^−1^ for Fe and *ca. *2425 µg l^−1^ for Sn at the end of the experiment. When normalized by photoelectrode area, the resulting values (*ca. *18 µg l^−1^ cm^−2^ and *ca. *49 µg l^−1^ cm^−2^, respectively) fall short compared to the values found in the literature for visibly aged α‐Fe_2_O_3_ photoelectrodes (e.g., 282 µg_Fe_ l^−1^ cm^−2^ [80]) and for FTO layers (e.g., 66 µg_Sn_ l^−1^ cm^−2^ [81]), confirming their relative stability in this work. This remarkable result highlights the physicochemical stability of the optimized micro‐scale substrate features and the FTO current collectors. The latter is especially relevant when compared to the metallic prone‐to‐corrosion current collectors (e.g., Ni [22], Ag [60], Au [38], or Cu [39] grids) commonly employed in large‐area PEC photoelectrodes – Table 1. Representative metal‐oxide photoelectrodes using such metallic collectors typically exhibit stability times ranging from only a few minutes to several tens of hours (≤80 h), even when mitigation strategies such as encapsulation [37] or partial coverage by the photoabsorber are employed. In contrast, the proposed FTO‐based current collectors enable a 10‐fold improvement in stable operational time.

## 3 Conclusions

This work addresses the most critical upscaling challenges observed in PEC‐WS systems by engineering upscaled substrates that effectively bridge the performance gap between small‐ and large‐area photoelectrodes. This performance loss is related, among other factors, to the low electronic conductivity of the substrates used in the preparation of photoelectrodes, which in the present study were FTO‐coated glass. This work then proposes a scalable deposition procedure to produce micrometre‐thick FTO line‐shaped current collectors, whose design and number were optimized using a conductivity simulator. The best‐performing design (consisting of 4 vertical/horizontal lines) reduced to less than half the ohmic losses imposed by a commercial 49 cm^2^ FTO‐coated glass substrate. In the following step, a glass texturization procedure was developed to produce micro‐structured features that increase the active surface area, while minimizing light transmittance and wettability. The optimized micro‐structured pattern was capable of increasing the surface area by >35%.

Both substrate engineering solutions were then combined to produce a 49 cm^2^ FTO‐coated glass substrate with increased conductivity and surface area. The optimized FTO‐glass substrate was then used to prepare a thin‐film α‐Fe_2_O_3_ photoelectrode, which was tested for the photo‐oxidation of water in an alkaline electrolyte. The concentration and flow rate of said electrolyte were adjusted to minimize ionic, concentration polarization, and pH gradient losses. The large photoelectrode produced *ca. *0.63 mA cm^−2^ at 1.45 V_RHE_ (100 mW cm^−2^), surpassing by *ca. *80% the photocurrent observed for a collector‐less flat surface reference and matching the performance observed in a similar small‐area system (*ca. *0.28 cm^2^). The stability of this photoelectrode was evaluated in a 1000 h chronoamperometry experiment under different simulated sunlight conditions and demonstrated full photocurrent stability. This impressive outcome underscores the physicochemical stability of the optimized micro‐scale substrate features and FTO current collectors. Notably, the FTO collectors exhibit over 10 times the stability of the corrosion‐prone metallic materials typically used in literature for similar purposes.

## 4 Theory

### 4.1 Simulation Details—FTO Current Collectors

The ohmic losses (or resistive overpotentials) across FTO‐coated glass substrates were simulated using COMSOL Multiphysics (Version 6.2), with the aid of the AC/DC Module (Electric Currents interface). This tool proved crucial in the optimization of the dimensions, relative positioning, and number of the proposed FTO line‐shaped current collectors [41]. The simulations began with the definition of the geometry under study. A rectangular prism was designed to replicate the dimensions of the glass substrate for the envisioned photoelectrodes: 9 cm of width (*x* in 3D Cartesian coordinates), 9 cm of length (*y*), and 2.2 mm of thickness (*z*). On top of this, a 645 nm‐thick FTO layer was added to simulate the commercial TCO coating. The FTO line‐shaped current collectors, assumed to be deposited on top of the commercial FTO layer, were modeled as 5 µm‐thick conductive strips with defined geometry and position.

A 3D finite element method (FEM) was used to discretize the geometry into smaller elements, following different mesh generational algorithms (triangular for glass and tetrahedral for the commercial FTO and FTO current collectors)—Figure S1. The electrical resistivity assigned to the commercial FTO was 4.5 × 10^−4^ Ω cm, calculated from the sheet resistance indicated by the supplier (TEC‐7, sheet resistance of *ca* 7 Ω □^−1^, Solaronix), and of 3.2 × 10^4^ Ω cm for the FTO current collectors (based on experimental measurements).

In a stationary coordinate system, the AC/DC conductive model of COMSOL uses a version of Ohm's Law to compute the electric currents in conductive media—Equation (1) [82]:

(1) J=σ×E

where *E = *(*Ex, Ey, Ez*) is the electric field (hence the bold‐face) and *σ* is the electrical conductivity. Under static conditions, the electric field is related to the electric potential (*V*) through Equation (2):

(2) E=−∇×V

where the charge conservation is guaranteed by the continuity equation—Equation (3).

(3) $$\nabla \times J=-\nabla \times \left(\sigma \nabla V\right)=0$$

Throughout the surface of the geometry, strategically placed point sources were considered for the current generation, using Equation (4).

(4) $$-\nabla \times \left(\sigma \nabla V\right)=Q_{j}$$

where *Q*_j_ is the boundary current source (set to 1 mA in this work). A constant potential boundary condition was applied to the perimeter edge of the substrates so as to simulate an electrical contact. In this zone, the potential was set to zero (see Figure S1a).

## 5 Experimental

### 5.1 Preparation and Characterization of Fluorine‐Doped Tin Oxide (FTO) Current Collectors

**FIGURE 11:**
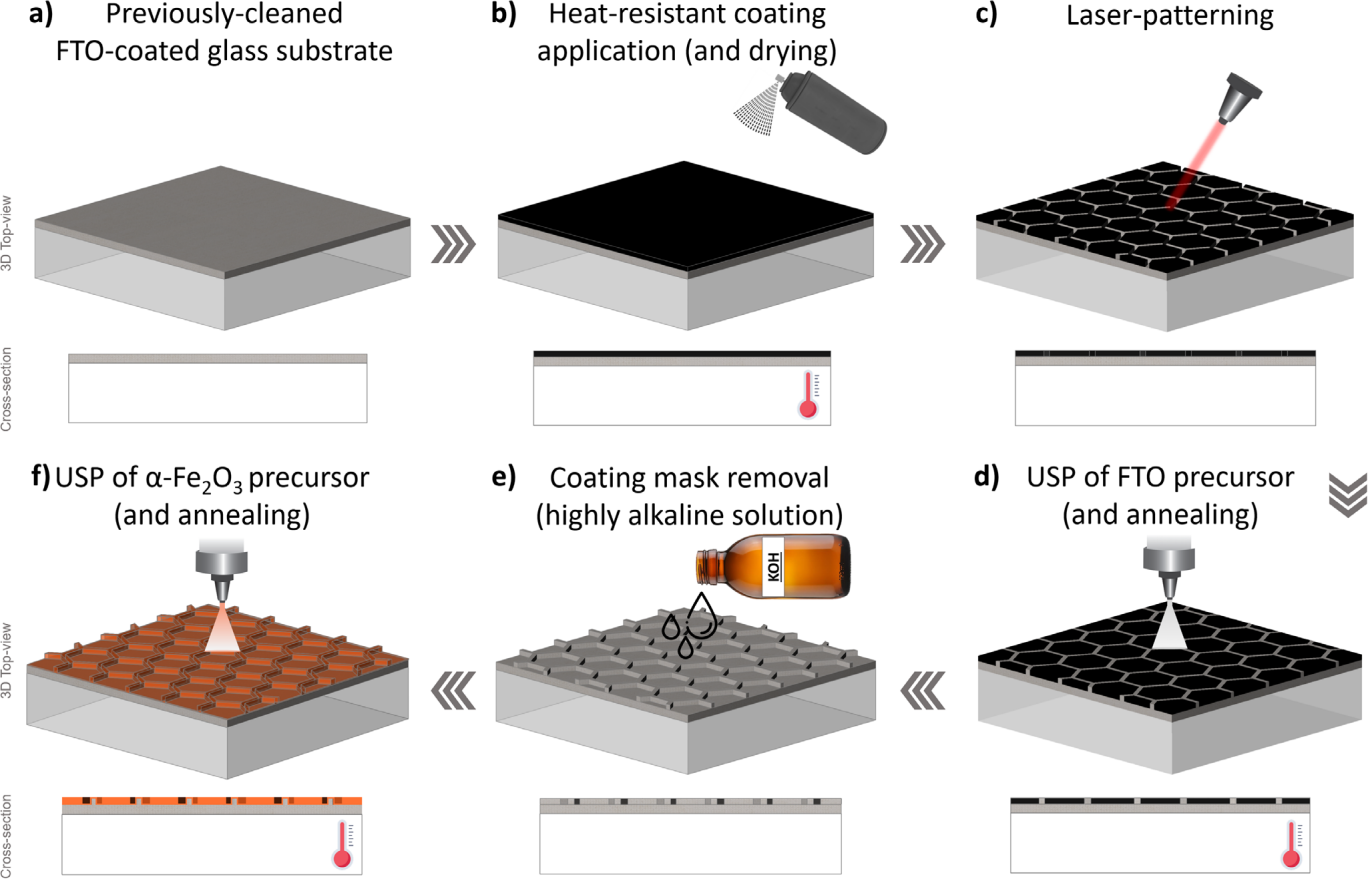
Schematic illustration of the lift‐off lithography‐assisted USP procedure used in the preparation of line‐shaped FTO current collectors.

Line‐shaped current collectors made of FTO were prepared using a lift‐off lithography‐assisted ultrasonic spray pyrolysis (USP) procedure recently proposed by the authors of this work [41]. Commercial FTO‐coated glass substrates (9 × 9 cm^2^, TEC‐7; 2.2 mm thick, 7 Ω □^−1^, Solaronix) were first thoroughly cleaned in distilled water and in a potassium hydroxide (KOH) aqueous solution [83]. The substrates were then spray‐coated with a heat‐resistant ink (Anthracite ink, Grouht) and cured for 2 h at 150 °C—Figure 11b. Afterward, the desired pattern for the current collectors was then defined via selective (thermal) ablation of the masking layer using pulsed laser ablation (350 nm, 5 W, LaserMaq) ‐ Figure 11c. The patterned substrates were then placed on a heating plate, below an ultrasonic spray nozzle, and heated to 450 °C. During the deposition procedure, the spray nozzle (25 kHz, ExactaCoat OP3, Sono‐Tek) moved over the sample's area at a speed of 10 mm s^−1^, while a syringe pump (Cronus Sigma) delivered the FTO precursor solution at a flow rate of 1 mL min^−1^ – Figure 11d. The shaping gas flow rate was set to 3 mL min^−1^, with a nozzle‐substrate distance of 10 cm and a nozzle power of 3 W. A total of 120 spray passages of precursor solution were performed, followed by an annealing step at 450 °C for 30 min. The FTO precursor solution consisted of 0.2 m tin (IV) chloride pentahydrate (SnCl_4_·5H_2_O, 98%, Thermo Scientific) dissolved in gradient‐grade absolute ethanol (EtOH, ≥99.9%, LiChrosolv, Sigma‐Aldrich), with ammonium fluoride (NH_4_F, 96%, Alfa Aesar) used as dopant, at a molar F/Sn ratio of 1:4. Following the USP deposition, the heat‐resistant mask was removed by immersion in a 10 vol% aqueous KOH solution at 50 °C for 8 h—Figure 11e.

The surface topography and film thickness of the line‐shaped FTO current collectors were examined using a high‐resolution scanning electron microscope (SEM, Quanta 400 FEG, FEI Company, available at CEMUP—Centro de Materiais da Universidade do Porto). Two‐point probe (2PP) resistance measurements were used to evaluate the sample's electrical resistance, using a source measurement unit (2425c SourceMeter, Keithley) and gold‐coated steel oscilloscope probes (PTR HARTMANN).

### 5.2 Preparation of Micro‐Structured FTO‐Coated Glass Substrates

The micro‐structuring of FTO‐coated glass substrates was made using a unique top–down experimental procedure that combines laser ablation‐assisted lithography, chemical etching, and spray pyrolysis techniques – Figure 5. In short, previously cleaned commercial FTO‐coated glass substrates (small – 1.2 × 3 cm^2^ or large – 9 × 9 cm^2^) were first positioned below a laser system capable of pulsed (thermal) ablation. Then, the power (0.8 W), frequency (20 kHz), and scanning speed (>400 mm s^−1^) of the laser head were optimized to selectively remove micrometre‐scale dot‐shaped portions of the FTO layer—Figure 5b. The laser path was programmed to create dot‐shaped ablated regions arranged in a 2D square matrix of 1000 × 1000 points. By adjusting the dimensions of this square‐shaped area of work, while keeping the number of pulses constant, it was possible to change the relative position and distance of each individual point/dot. Following laser treatment, the patterned substrates were periodically immersed in a buffered oxide etchant (BOE) composed of a 6:1:1 ratio (v/v/v) of ammonium fluoride (NH_4_F, 99.9%, Sigma‐Aldrich), hydrofluoric acid (HF, 40%, Sigma‐Aldrich), and hydrochloric acid (HCl, 37%, Sigma‐Aldrich). The BOE selectively etched the exposed soda‐lime glass, forming groove‐like microcavities at a rate of *ca. *0.3 µm s^−1^ (45 °C), which further increased the surface roughness and effective area of the substrate—Figure 5c. In the last step of the preparation procedure, a thin FTO layer was spray‐coated onto the substrates to restore the electrical conductivity lost during patterning and etching—Figure 5d. As described elsewhere [24], the deposition started by placing the substrates on a heating plate and heating them to *ca. *490 °C, with the temperature measured at the surface of the sample. A glass‐atomizer (12 mL, Lenz Laborglas GmbH & Co. KG) was then used to spray discrete volumes of a precursor solution containing tin(II) chloride dehydrate (SnCl_2_·2H_2_O, 98%, Sigma‐Aldrich), NH_4_F, absolute EtOH, distilled water, and HCl. An optimized H_2_O/EtOH volume ratio of 0.43 and a [F]/[Sn] ratio of 0.45 were employed. The atomizer was fixed *ca. *20 cm above the substrate, and an automated compressed‐air dispenser (DC200, Fisnar) was employed to guarantee the reproducibility between each spray. During the deposition procedure, several sets of 10 consecutive sprays were performed, with a time gap of 20 s, until *ca. *42 mL of precursor solution was spent. Finally, the substrates were air‐annealed for 30 min at the deposition temperature. Each sample was labeled according to its laser‐processed area and etching time, as highlighted in Table 3.

### 5.3 Characterization of Micro‐Structured FTO‐Coated Glass Substrates

Small‐scale micro‐structured FTO‐coated glass substrates were first characterized in a three‐electrode electrochemical setup by EIS. The measurements were performed in a “cappuccino” (photo)electrochemical cell [84], allowing an illumination area of *ca. *0.28 cm^2^. The working electrode consisted of the micro‐structured substrate; a platinum wire (Alfa Aesar) served as the counter electrode, and an Ag/AgCl/Sat. A KCl electrode (Metrohm) was used as the reference electrode. All electrodes were immersed in 1 M KOH aqueous solution (VWR, room temperature, pH = 13.5). Finally, an AUTOLAB electrochemical station (PGSTAT302N) was used to supply a sinusoidal potential perturbation with an amplitude of 10 mV (centered at the open‐circuit potential—*E*_OCP_) and record the system's response over a frequency range of 0.1 Hz to 100 kHz. The low‐frequency region of the impedance spectra was fitted to an equivalent circuit model using ZView software (version 3.5d, Scribner Associates, Inc.) consisting of: (1) a series resistance (*R*_s_) related to ohmic (at the FTO and electric wiring) and ionic (at the electrolyte) resistances of the system; and (2) a Randle's circuit that describes charge transfer (charge‐transfer resistance—*R*_ct_) and charges accumulation (double‐layer capacitance—*C*_dl_) at the electrode–electrolyte interface [85] ‐ Figure S7. This work uses the *C*_dl_ estimated by EIS at the *E*_OCP_ (where little to no Faradaic charge‐transfer processes take place) as a way to quantitatively estimate the surface area gain resulting from the micro‐structuring procedure.

The optical transmittance of the proposed micro‐structured substrates was measured using a UV–vis–NIR spectrophotometer (model UV‐3600, Shimadzu Scientific Instruments Inc.). Four‐point probe (4PP) resistance measurements were used to estimate a pseudo‐sheet‐resistance ($R_{\mathrm{sheet}}^{\prime }$), applying the van der Pauw method [86]. The surface topography was analysed by SEM and by atomic‐force microscopy (AFM). For the latter, a NanoScope Multimode Atomic Force Microscope (Veeco Instruments Inc.) was used in tapping mode (TESP‐V2 probe, Bruker) to estimate the root‐mean‐square surface roughness (*R*_q_, in nm) of the FTO layer inside and outside the etched micro‐grooves. Finally, contact angle measurements were performed to evaluate the wettability to water and EtOH of the proposed micro‐structured surfaces. During this test, an optical contact angle measuring equipment (OCA 20, DATAPHYSICS), coupled with an automatic dosing system (syringes: Hamilton 500 µL for water and Braun 1 mL for EtOH), deposited a drop of liquid on the surface of the substrate with a stipulated volume (2 µL for water and 4 µL for EtOH). The contact angle was determined from captured images using the SCA20 software (DATAPHYSICS).

### 5.4 Preparation of Hematite Photoelectrodes

Ultrathin bare hematite (α‐Fe_2_O_3_) photoelectrodes were prepared by spray pyrolysis on the proposed high conductivity and surface area FTO‐coated glass substrates. According to the size of the substrate, either a pneumatic or an ultrasonic spray head was employed for the deposition of the hematite precursor solution. For small‐area α‐Fe_2_O_3_ photoelectrodes, the deposition began by placing an FTO‐coated glass substrate on a heating plate, which was heated to 400°C. After a pre‐treatment with a tetraethyl orthosilicate (TEOS, 98%, Sigma‐Aldrich) solution (10 vol% in ethanol) [53], an ultrasonic spray nozzle (25 kHz, ExactaCoat OP3, Sono‐Tek, *ca. *5 cm above the plate) was moved across the substrates at a constant speed of 10 mm s^−1^, continuously spraying a 10 mm solution of Fe(acac)_3_ (iron (III) acetylacetonate, 99.9%, Sigma‐Aldrich) in EtOH (flow rate of 1 mL min^−1^). A shaping gas flow rate was set to 2 mL min^−1^, and the nozzle power was 2 W. A total of 15 spray passages were performed, consuming *ca. *4 mL of precursor solution. This yielded dry α‐Fe_2_O_3_ films with a thickness of *ca. *20–30 nm (estimated by UV–visible absorption data) [87].

For large‐area depositions (substrates of 9 × 9 cm^2^, with an effective deposition area of 7 × 7 cm^2^), the procedure followed a previously established protocol [15]. First, a conductive silver paste line (Ferro GmbH GSSP SP 1963) was manually printed on both sides of the substrate edges and sintered at 500 °C. This metallic frame created a conductive path between both sides of the substrate, making the current collection more efficient during photoelectrochemical characterizations [64]. The substrate was then placed on a heating plate, below a pneumatic spray nozzle, and TEOS‐treated at 450 °C. An automatic syringe pump (Cronus Sigma 2000 Dual, LabHut) later delivered discrete volumes (1 mL) of 10 mm Fe(acac)_3_ precursor solution at a flow rate of 12 mL min^−1^. The time interval between each spray was 35 s, and a total volume of 70 mL of solution was fed to the nozzle. A final annealing was performed for 30 min at 500 °C.

### 5.5 Characterization of the Hematite Photoelectrodes

All small‐area α‐Fe_2_O_3_ photoelectrodes were characterized in a “cappuccino” PEC cell using the three‐electrode experimental setup previously described. Current density versus potential curves (*J‐*‐*E*) were recorded using an AUTOLAB electrochemical station. Measurements were performed at room temperature, both in the absence (dark) and presence (light) of a simulated solar illumination of 100 mW cm^−2^ (1‐sun, calibrated with a c‐Si photodiode, Newport). The light source was a class B solar simulator (150 W Xenon lamp, Oriel, Newport) with an AM 1.5G filter. The external bias was applied at a scan rate of 10 mV s^−1^ and reported as a function of the reversible hydrogen electrode (RHE).

Large‐area α‐Fe_2_O_3_ photoelectrodes were characterized by *J‐*‐*E* measurements in a “PortoCell” PEC device (photoactive area of 7 × 7 cm^2^) [43]. To perform such tests, the photoelectrode was first mounted as the window of the device and connected to the electrochemical station as working electrode. The counter electrode was a platinized Ti mesh placed in a back‐to‐back configuration relative to the working electrode, with an Ag/AgCl/Sat KCl reference electrode in between (Figure S4). Unless stated otherwise, the electrolyte used consisted of a commercial 1 M KOH aqueous solution. *J*–*E* measurements were performed as previously described for small‐scale photoelectrodes, both in the dark and under simulated solar illumination (sulfur plasma lamp, system AS 1300 V 2.0, Plasma International GmbH, calibrated with a c‐Si photodiode). Whenever relevant, data acquired from the *J*–*E* curves were used to estimate the purely light/induced photocurrent *J*_photo_, photopotential *V*_photo_, and the fill‐factor (FF) as detailed in Section S3.

### 5.6 Test Bench for the Long‐Term Stability Test

For assessing the long‐term stability and performance of a thin‐film α‐Fe_2_O_3_ photoelectrode prepared using the proposed optimized substrate, a dedicated stability test bench was designed. After assembling the photoelectrode, counter, and reference electrodes in the “PortoCell”, the device was then mounted on a metallic support and aligned with the solar simulator (sulfur plasma lamp, system AS 1300 V 2.0, Plasma International GmbH). Afterward, electrolyte recirculation (50 mL min^−1^) was promoted by a peristaltic pump (323 series, Watson Marlow), employing Teflon tubing (internal diameter, *ø*_int_ = 4 mm) to connect the cell and the reservoir. The reservoir consisted of a double‐walled borosilicate glass bottle (500 mL, GLS 80, Duran), filled with 500 mL of 4 M KOH aqueous solution. During the test, an AUTOLAB electrochemical station measured the photogenerated current at 1.45 V_RHE_, as the photoelectrode was illuminated using the solar simulator. To prevent device overheating under extended solar exposure, an aluminum thermal shield was assembled (Figure S4). The electrolyte temperature was monitored in real‐time and maintained below *ca. *32 °C with the aid of a thermostatic bath (F12 EH, Julabo).

After the stability experiment, the surface of fresh and aged α‐Fe_2_O_3_ thin films was characterized by SEM, energy‐dispersive X‐ray spectrometry (EDS, EDAX Genesis X4M), and by X‐ray photoelectron spectroscopy (XPS, ESCALAB 250Xi, Thermo Fisher Scientific). The XPS measurements were performed using a monochromated Al *K*_α_ X‐ray source (*hv* = 1486.68 eV), operated at 220 W, 14.6 kV, and with a spot size of 650 µm. A hemispherical analyser with energy resolution of 0.1 eV was used, with survey and individual element spectra collected at pass energies of 100 eV and 40 eV, respectively. The XPS spectra were peak‐fitted using the CasaXPS software. Throughout the stability test, electrolyte samples of *ca* 1.5 mL were periodically collected and stored at room temperature. Later, inductively coupled plasma‐optical emission spectroscopy (ICP‐OES, iCAP 7000 series, Thermo Fisher Scientific) was used to investigate the release of iron (Fe) and tin (Sn) from the photoelectrode onto the electrolyte.

## Funding

The research leading to this work has received financially support by national funds through the FCT/MCTES (PIDDAC) under the project ASAPFuels—PTDC/EQU‐EQU/4225/2021 (DOI:10.54499/PTDC/EQU‐EQU/4225/2021) and COMPETE/FCT under the project DreamPEC COMPETE2030‐FEDER‐00898000, and through the FCT/MECI by LEPABE, UID/00511/2025 (https://doi.org/10.54499/UID/00511/2025) and UID/PRR/00511/2025 (https://doi.org/10.54499/UID/PRR/00511/2025), by ALiCE—LA/P/0045/2020 (DOI: 10.54499/LA/P/0045/2020) and by CONSTRUCT—UIDB/04708/2020 (DOI: 10.54499/UIDB/04708/2020).

## Conflicts of Interest

The authors declare no conflict of interest.

## Supporting information




**Supporting File**: advs73782‐sup‐0001‐SuppMat.docx.

## Data Availability

The data that support the findings of this study are available in the supplementary material of this article.
