# Supplementary material for: Highly‐Conductive and Micro‐Structured Transparent Glass Substrates for Efficient and Scalable Photoelectrochemical Applications
Source: Adv Sci (Weinh). 2026 Apr 9;13(28):e15947. doi: 10.1002/advs.202515947 (PMC13185818; doi:10.1002/advs.202515947)
Supplement: Supplementary file 1 — Supporting File: advs73782‐sup‐0001‐SuppMat.docx. [file ADVS-13-e15947-s001.docx]

Supporting Information

**Highly-conductive and micro-structured transparent glass substrates for efficient and scalable photoelectrochemistry**

*Telmo da Silva Lopes, Jeffrey Capitão, Amin Khan, Leonardo Rodrigues, Dzmitry Ivanou, Tânia Lopes, Paula Dias*, Adélio Mendes*

T. da Silva Lopes, J. Capitão, A. Khan, L. Rodrigues, D. Ivanou, T. Lopes, P. Dias, A. Mendes:

LEPABE – Laboratory for Process Engineering, Environment, Biotechnology and Energy, Faculty of Engineering, University of Porto, Rua Dr. Roberto Frias, 4200-465 Porto, Portugal;

ALiCE – Associate Laboratory in Chemical Engineering, Faculty of Engineering, University of Porto, Rua Dr. Roberto Frias, 4200-465 Porto, Portugal

E-mail address: [pauladias@fe.up.pt](mailto:pauladias@fe.up.pt), Tel.: +351 225081695; Fax: +351 225081449;

L. Rodrigues:

CONSTRUCT-LFC, Department of Civil Engineering, Faculty of Engineering, University of Porto, 4200-465 Porto, Portugal

**S1. Experimental details of representative demonstrations of upscaled photoelectrochemical (PEC) water splitting**

**Table S1.** Extensive review of upscaled semiconductor-liquid junction PEC water splitting (PEC-WS) demonstrations divided by photoabsorber material and ordered by date of publication.

| **Area^a)^ in small \| large substrates / cm^2^** | **Photoelectrode**  **(Substrate)** | **Photocurrent density in small \| large substrates / mA·cm^-2^** | **Operation time (of large-area demo)** | **Year, Ref.** |
| --- | --- | --- | --- | --- |
| *BiVO_4_: Bismuth vanadate-based photoelectrodes* | | | | |
| 16000 | Mo-doped BiVO_4_/Co-Pi (FTO-coated glass) in series with Si-HTJ PV cells | N/A \| 3.00^b)^  (@0 V, AM 1.5G, 100 mW·cm^-2^) | 1000 h in outdoor | 2017[1] |
| 2 \| 25 | WO_3_/Mo-BiVO_4_/Co-Pi (FTO-coated glass) | 2.20 \| 0.74  (@1.23 V_RHE_, AM 1.5G,  100 mW·cm^-2^) | 1 h^b)^ | 2018[2] |
| 1 \| 300 | Metal-doped BiVO_4_  (FTO-coated glass) | 1.07 \| 0.22  (@1.23 V_RHE_, AM 1.5G,  100 mW·cm^-2^) | 4 h | 2018[3] |
| 1 \| 56 | BiVO_4_/NiOOH  (FTO-coated glass) | 2.20 \| 0.18^b)^  (@1.23 V_RHE_, AM 1.5G,  100 mW·cm^-2^) | 4 h | 2020[4] |
| 1 \| 25 \| 225 | BiVO_4_/NiFeO_x_  (FTO-coated glass) | 4.40 \| 2.10 \| 1.30  (@0.6 V_RHE_, AM 1.5G,  100 mW·cm^-2^) | 135 h (25 cm^2^) \| 1 h every 3 days (225 cm^2^) | 2020[5] |
| 0.24 \| 50 | W:BiVO4/Co-Pi  (FTO-coated glass with Ni grid) in series with Si-HTJ PV cells | 4.50 \| 1.70  (@0 V, AM 1.5G,  100 mW·cm^-2^) | 10 min | 2020[6] |
| <1 \| 41.18 | BiVO_4_/NiFeOOH  (FTO-coated glass with Au current collector) | 2.80 \| 2.20  (@0.6 V_RHE_, AM 1.5G,  100 mW·cm^-2^) | ≈ 5 h | 2020[7] |
| 1 \| 100 | BiVO_4_/TiCo  (Ti foil) Artificial leaf with Lead halide perovskite photocathode | 0.60 \| 0.20^b)^  (@0 V, AM 1.5G, 100 mW·cm^-2^) | 18 h | 2022[8] |
| 1 \| 25 | TD-BiVO_4_^c)^  (FTO-coated glass) in tandem with Cu_2_ZnSnS_4_ photocathode | 6.60^b)^ \| 0.60  (@0 V)^b), c)^ | 7 × 8 h | 2022[9] |
| 1 \| 100 | W:BiVO_4_/NiFeOOH  (Ti felt) in series with Si PV cell | 2.30 \| 2.10  (@0 V, AM 1.5G, 100 mW·cm^-2^) | 6 h | 2023[10] |
| 0.07 \| 36 | Au-decorated WO_3_/BiVO_4_ nanorods (FTO-coated glass) | 2.80^b)^ \| 1.20^b)^  (@1 V_RHE_, AM 1.5G,  100 mW·cm^-2^) | N/A | 2023 [11] |
| NA \| 25 | WO_3_/BiVO_4_/Co-Pi  (FTO-coated glass with Ni grid) | 3.60^b)^ \| 2.80  (@1.23 V_RHE_, AM 1.5G,  100 mW·cm^-2^) | 80 h | 2023[12] |
| 1 \| 25 | BiVO_4_  (FTO-coated glass with Ag grid) | 0.89 \| 0.61  (@1.23 V_RHE_, AM 1.5G,  30 mW·cm^-2^) | 10 h | 2023[13] |
| *α-Fe_2_O_3_: Hematite-based photoelectrodes* | | | | |
| N/A \| 100^b)^ | α-Fe_2_O_3_/TiO_2_  (Ti sheet) | N/A \| 0.12^b)^  (@0 V_HgO\|Hg_, 42 mW·cm^-2^) | N/A | 2013[14] |
| N/A \| 100 | α-Fe_2_O_3_ functionalized with Phycocyanin  (FTO-coated glass) | N/A \| 0.45^b)^  (@1.45 V_RHE_, AM 1.5G,  100 mW·cm^-2^) | N/A | 2014[15] |
| <1 \| 100 | α-Fe_2_O_3_  (FTO-coated glass) | 0.63^b)^ \| 0.40  (@1.45 V_RHE_, AM 1.5G,  100 mW·cm^-2^) | N/A | 2014[16] |
| N/A \| 100 | α-Fe_2_O_3_  (Ti sheet) | N/A \| ≈ 0.33^b)^  (@1.50 V_RHE_, AM 1.5G,  100 mW·cm^-2^) | N/A | 2016[17] |
| 0.5^b)^ \| 50 | α-Fe_2_O_3_  (FTO-coated glass) | ≈ 0.94^b)[18]^ \| ≈ 0.48^b)^  (@1.45 V_RHE_, AM 1.5G,  100 mW·cm^-2^) | 1008 h | 2018[19] |
| 3.2 \| 8 × 3.2 | α-Fe_2_O_3_  (FTO-coated glass) | 0.65  (@1.45 V, AM 1.5G,  100 mW·cm^-2^) | N/A | 2018[20] |
| N/A \| 100 | α-Fe_2_O_3_  (FTO-coated glass) in tandem with Si-based photovoltaic | N/A \| 0.55  (@0 V, AM 1.5G, 100 mW·cm^-2^) | 10 × 8.3 h | 2020[21] |
| 5.5 \| 4 × 8 × 5.5 | Worm-like nanostructured α-Fe_2_O_3_  (FTO-coated glass) | ≈ 0.75^b)^ \| ≈ 0.50^b)^  (@1.45 V, real sunlight,  ≈ 100 mW·cm^-2^) | 48 h | 2020[22] |
| 0.25 \| 40 | α-Fe_2_O_3_  (FTO-coated glass) in tandem with CuO photocathode | -1.10 \| ≈ -0.67  (@-0.6 V, AM 1.5G, 100 mW·cm^-2^) | ≈ 5 h | 2024[23] |
| N/A \| 8000^b)^ | α-Fe_2_O_3_  (drilled FTO-coated glass in assembly with a hydrophobic diffusion layer membrane) in tandem with CuO photocathode | -1.10^b)[23]^ \| ≈ -0.01  (@-0.6 V, real sunlight,  ≈100 mW·cm^-2^) | ≈ 6 h | 2023[24] |
| 0.28 \| 49 | **α-Fe_2_O_3_**  **(Micro-structured FTO-coated glass with FTO line-shaped current collectors)** | **0.63 \| 0.63**  **(@1.45 V_RHE_, 100 mW**·**cm^-2^)** | **≈ 1000 h** | **This Work** |
| *WO_3_: Tungsten trioxide-based photoelectrodes* | | | | |
| 0.36 \| 130 | WO_3_  (FTO-coated glass with embedded Ag grid) | 2.63 \| ≈ 1.18  (@1.23 V_RHE_, AM 1.5G,  100 mW·cm^-2^) | N/A | 2011[25] |
| N/A \| 81 | Multi-layered WO_3_  (Stainless steel substrate) | N/A \| ≈ 0.44  (@1.40 V_RHE_, AM 1.5G,  100 mW·cm^-2^) | 10 min | 2011[26] |
| N/A \| 100 | WO_3_  (W foil) | N/A \| 0.90  (@1.45 V_RHE_, AM 1.5G,  100 mW·cm^-2^) | N/A | 2014[16a] |
| N/A \| 2 × 7.1^b)^ | WO_3_/p^+^n Si  (FTO-coated glass) in tandem with Pt/TiO_2_/Ti/n^+^pSi photocathode | N/A \| ≤ 1.35^b)^  (AM 1.5G, 200 mW·cm^-2^) | 20 h | 2015[27] |
| *TiO_2_: Titanium dioxide-based photoelectrodes* | | | | |
| 1 \| 40 | LaTiO_2_N/ Particles of NiO_x_/CoO_x_  (FTO-coated glass) | 2.29 \| 0.56  (@1.23 V_RHE_, AM 1.5G,  100 mW cm^-2^) | 110 min | 2019[28] |
| N/A \| 10 | TiO_2_  (Ti sheet) | N/A \| 0.92^b)^  (@1.23 V_RHE_, AM 1.5G,  100 mW·cm^-2^) | 6 h | 2020[29] |
| *Cu_x_O: Cuprous oxide-based photoelectrodes* | | | | |
| 0.25 \| 50 | Cu_2_O/AZO/RuO_x_  (FTO-coated glass with line-shaped Cu grid) | 5.10 \| 3.10  (@0 V_RHE_, AM 1.5G,  100 mW·cm^-2^) | 60 min | 2021[30] |
| *Ta_3_N_5_: Tantalum nitride-based photoelectrodes* | | | | |
| 2 \| 6.25 | Ta_3_N_5_/Co-Pi  (Ti sheet) | 3.00 \| 3.00  (@1.23 V_RHE_, AM 1.5G,  100 mW·cm^-2^) | 120 min | 2019[31] |
| *Others* | | | | |
| 2.25 \| N/A | Al_2_O_4_Zn  (FTO-coated glass with line-shaped Ag nanoparticles grid) | N/A \| N/A | N/A | 2021[32] |

a) The area considered is the active area of the photoabsorber material (s); b) Estimated or calculated from the reported data; c) Thermoelectric device (TD) was coupled to the PEC cell to enhance the photo-induced photopotential. Small-area PEC system was tested indoors under AM 1.5G, 100 mW cm^-2^ (@0V), while the large-area substrates were tested outdoors with natural seawater.^[9]^ N/A – information not available at the source report.

**S2. Additional information on the conductivity simulations**


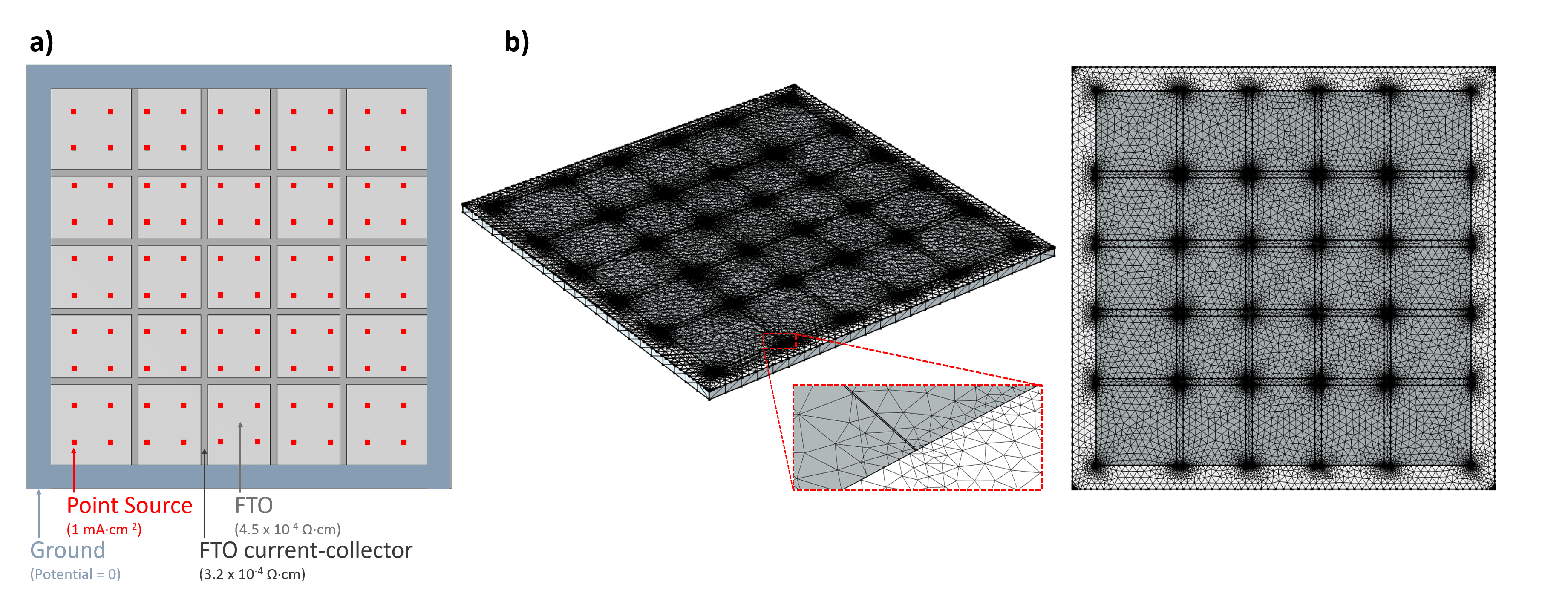


**Figure S1.** A 3D geometry was used to simulate the electrical conductivity and to optimize the design pattern of the proposed FTO current collectors: a) top view, where the red dots represent point sources for the current generation, and the blue area represents the ground; b) Isometric (left) and top views (right) of the mesh.


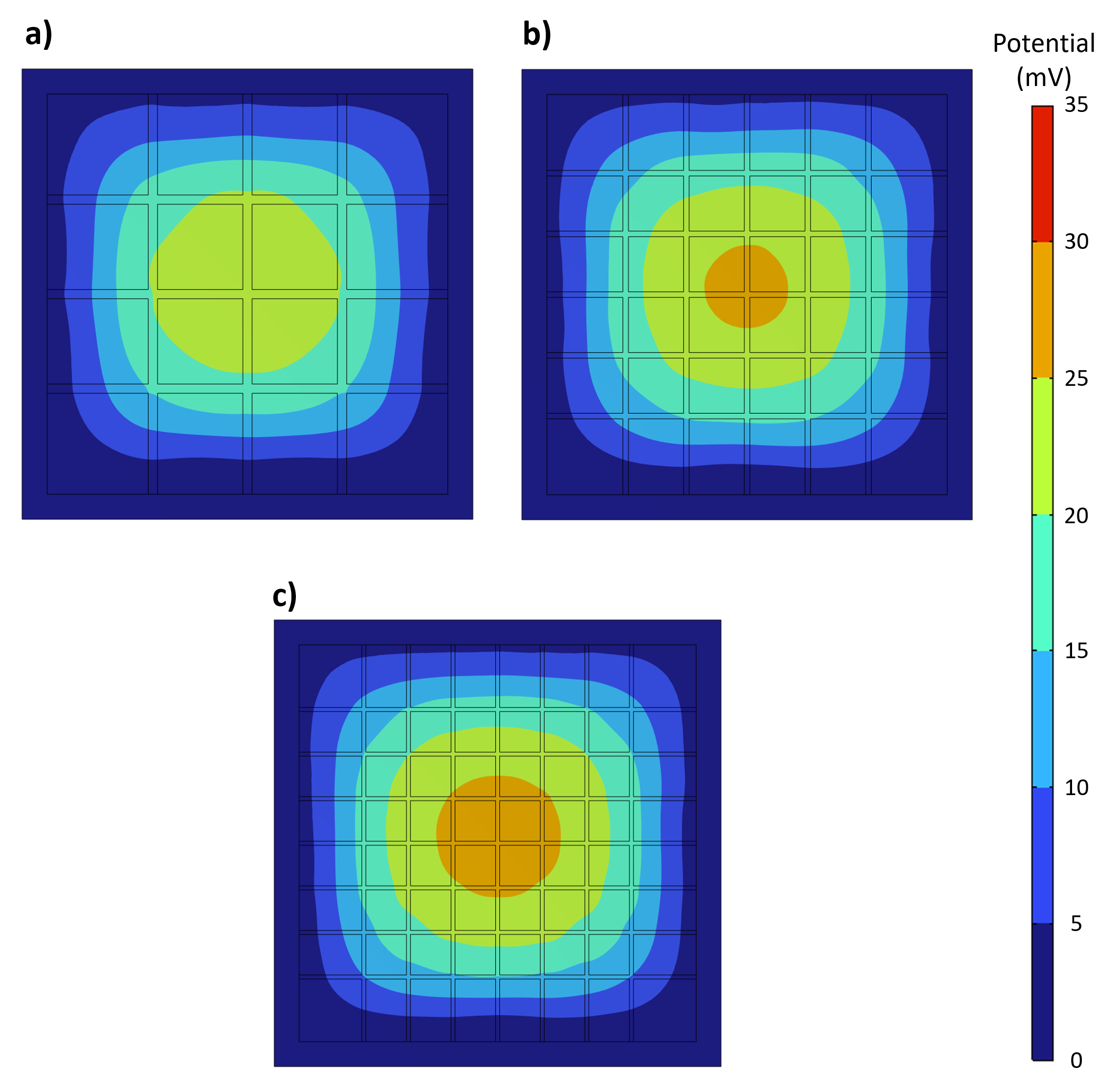


**Figure S2.** Overpotential contours calculated using the proposed conductivity model and the upscaled substrates under study for FTO-coated glass with: a) 3-; b) 5-; and c) 7-horizontal/vertical FTO current collectors.

**S3. Additional information on the characterization of large-area photoelectrodes**

*Calculation of the power characteristics of the photoelectrodes*

Data acquired from the *J*-*E* curves measured on large-area photoelectrodes was used to estimate their power characteristics. Following a procedure described elsewhere^[33]^, the purely light-induced photocurrent *J*_photo_ and photopotential *V*_photo_ were calculated from the measured light/dark data (*J*_light/dark_ and *V*_light/dark_) – Equation (S1) and (S2).

$J_{\mathrm{photo}} = J_{\mathrm{light}}- J_{\mathrm{dark}}$ (S1)

$V_{\mathrm{photo}}\left( J_{\mathrm{photo}} \right) = V_{\mathrm{dark}}\left( J_{\mathrm{photo}} \right)-V_{\mathrm{light}}\left( J_{\mathrm{photo}} \right)$ (S2)

By plotting *J*_photo_ as a function of *V*_photo_, an estimation of the short-circuit photocurrent (*J*_sc_) and the open-circuit photopotential (*E*_OCPh_) was obtained (Figure S3b). The product of *J*_photo_ and *V*_photo_ yielded the light-induced electric power (*P*) produced by the photoelectrode. The plot of *P* *vs* *V*_photo_ allowed the identification of the maximum power point photopotencial (*E*_MPPh_) and photocurrent (*J*_MPPh_) – Figure S3c. These parameters were used to estimate the fill-factor (FF) of the *J*_photo_ *vs* *V*_photo_ curve, as shown in Equation (S3).

$\mathrm{FF}\boldsymbol{=}\frac{E_{\mathrm{MPPh}}\cdot J_{\mathrm{MPPh}}}{E_{\mathrm{OCPh}} \cdot J_{\mathrm{SC}}}$ (S3)


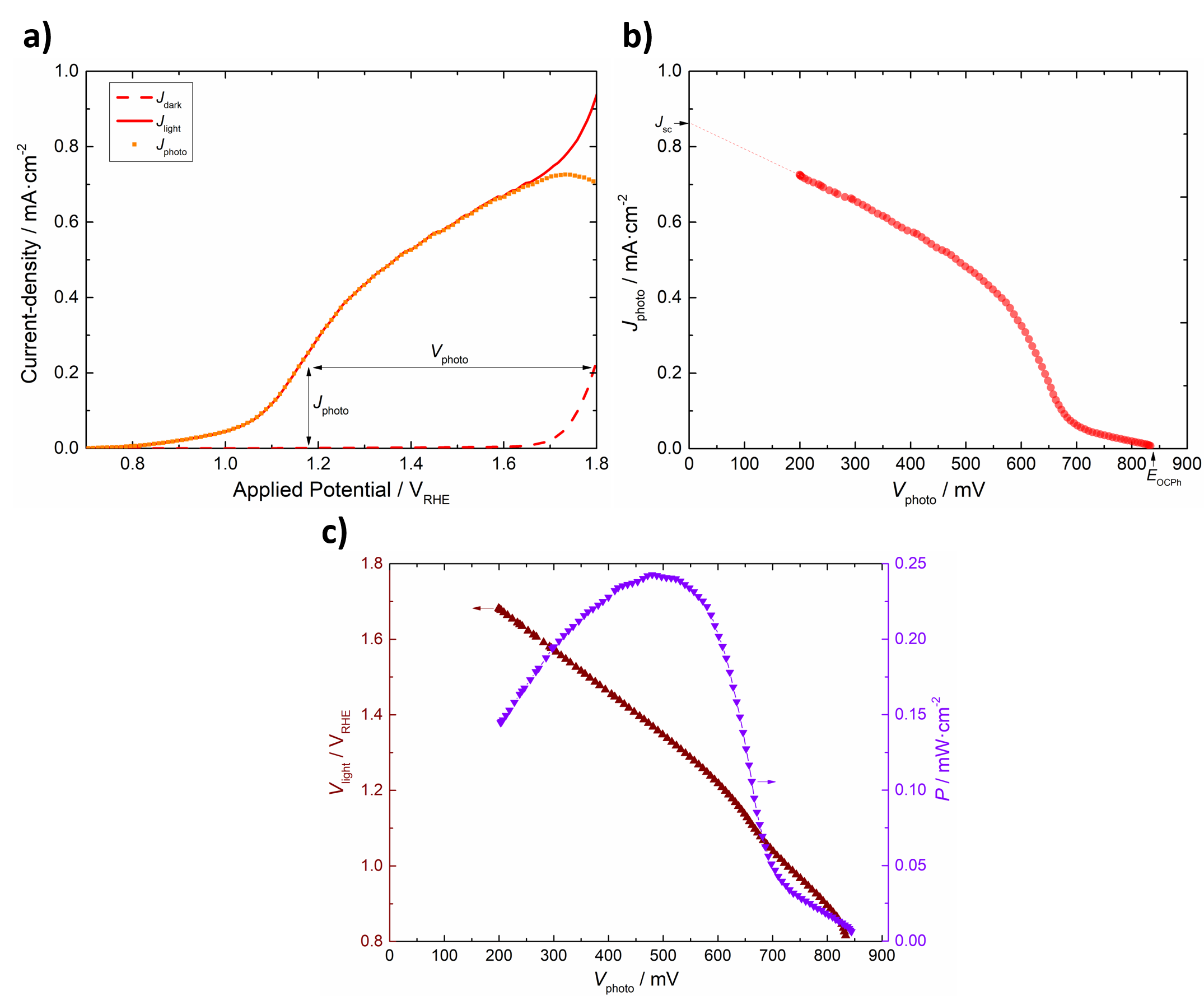


**Figure S3.** (a) Dark/Light (100 mW∙cm^-2^) J-E curves obtained for the bare α-Fe_2_O_3_ photoelectrode labeled in the main manuscript as 4-lines or A-CC and calculation of its light-induced photocurrent and photopotential (J_photo_ and V_photo,_ respectively); (b) J_photo_ vs V_photo_; (c) light-induced electric power produced by the photoelectrode (P) and V_light_ as a function of V_photo_.

*Details on the experimental setup*


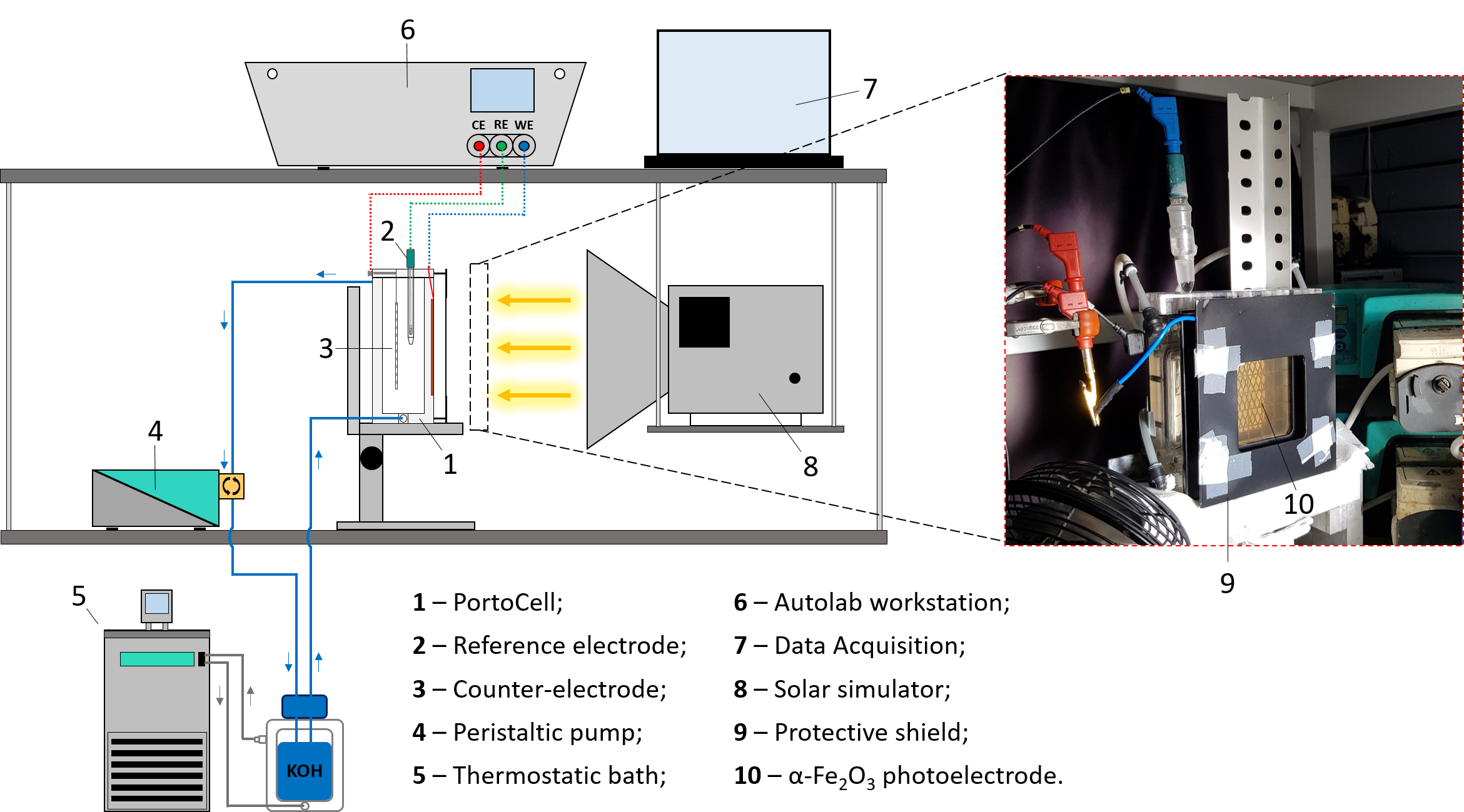


**Figure S4.** Experimental setup used in the characterization of the large-area photoelectrodes (PEs) used in this work: inset magnification of the positioning of a thin-film bare α-Fe_2_O_3_ photoelectrode and of the protective shield in the proposed PortoCell. The thermostatic bath and the protective shield were only utilized during the 1000 h long chronoamperometry test.

*Optimization of electrolyte concentration and flow-rate*

State-of-the-art PEC-WS devices equipped with α-Fe_2_O_3_ photoelectrodes use as electrolyte highly-alkaline solutions of potassium/sodium hydroxide (KOH/NaOH), typically 1 M. Taking as an example KOH, the ionic conductivity of aqueous solutions of this hydroxide shows a non-linear increase with concentration – Figure S5. At *ca.* 22 ºC, a 1 M solution has a conductivity of *ca.* 200 mS·cm^-1^, gradually rising to *ca.* 500 mS·cm^-1^ at 4 M KOH. From this concentration onward, conductivity plateaus or even decreases due to an ionic crowding effect.^[34]^ Figure S6a shows the photocurrent density at 1.45 V_RHE_ for the 49 cm^2^ A-CC-MSP photoelectrode (see main manuscript for the meaning of this label) and for the equivalent small-scale reference, both using aqueous solutions of KOH with different concentrations and with no electrolyte flow. As can be observed, an electrolyte concentration of 1 M seems to have enough conductivity to maximize the current density displayed by the small-scale system (*ca.* 0.28 cm^2^). However, for the larger system, a gradual performance increase is observed with electrolyte concentration, closely mimicking the solution’s conductivity pattern. The same test was repeated with the 49 cm^2^ A-CC-MSP photoelectrode, this time considering electrolyte flow at rates between 0-150 ml·min^-1^. As the analysis of Figure S6b shows, there is a clear performance dependence on flow-rate for KOH concentrations up to *ca.* 2 M. For this concentration onwards, this dependence decreases, suggesting that electrolyte conductivity is enough to provide the system with enough ionic mobility. Nevertheless, electrolyte flow is still necessary for continuous operation to allow the removal of the evolved gas and to minimize electrolyte concentration fluctuations.





**Figure S5.** Conductivity of aqueous solutions of KOH as a function of the molar concentration: experimental results (black square symbols, with dashed line) and values found in literature^[35]^ (red dashed line).

**
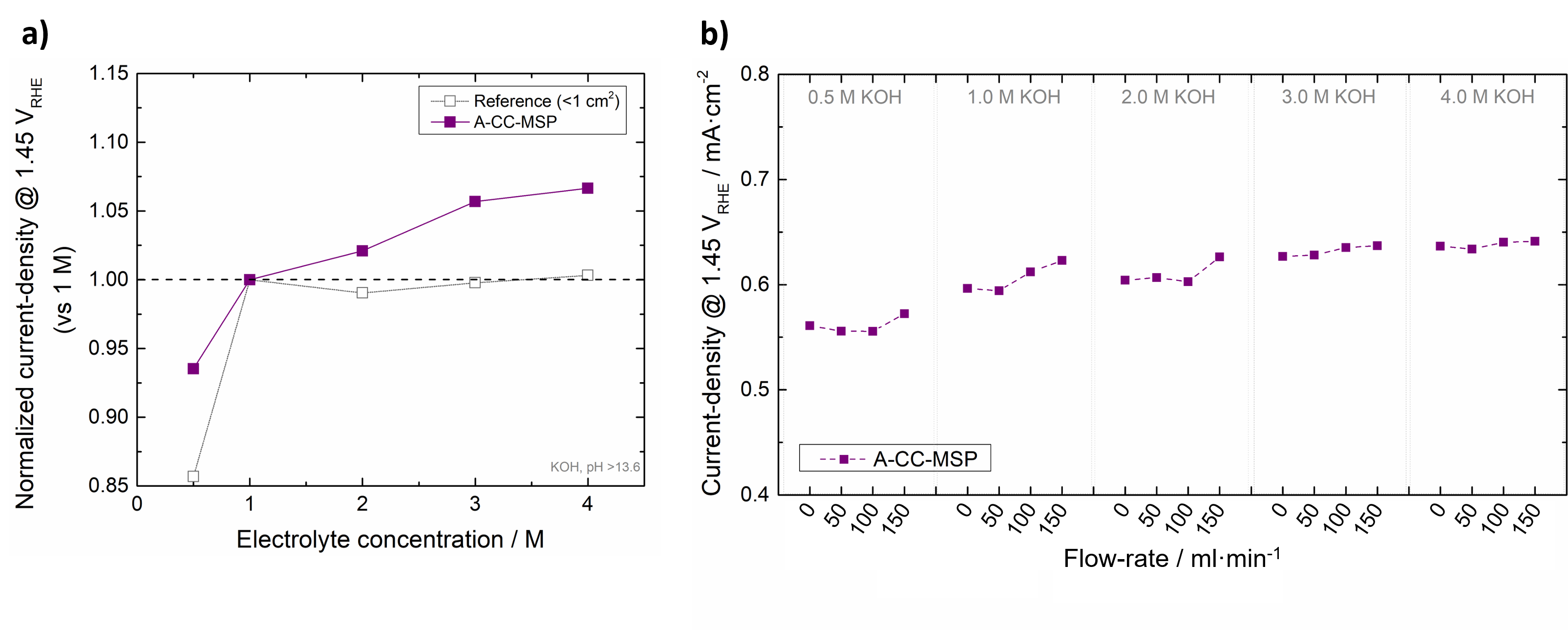
**

**Figure S6**. a) Normalized photo-generated current density (at 1.45 V_RHE_) for Reference (<1 cm^2^) and A-CC-MSP thin-film α-Fe_2_O_3_ photoelectrodes, as a function of electrolyte concentration (KOH, pH > 13.6) and in the absence of recirculation; b) photo-generated current density (at 1.45 V_RHE_) for A-CC-MSP as a function of electrolyte concentration and flow-rate.

**S4. Additional information on the optimization of micro-structured patterns for FTO-coated glass substrates**

***
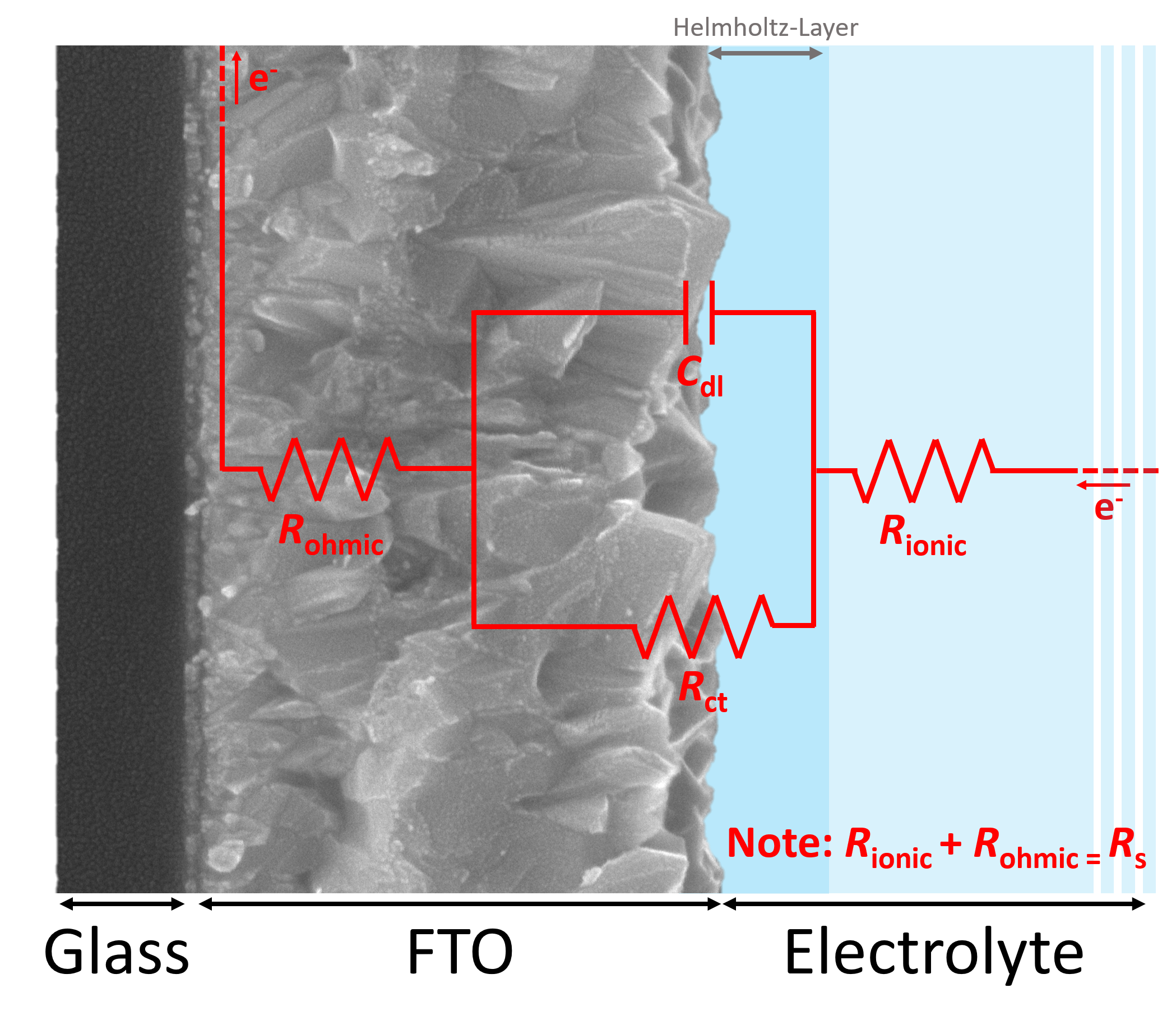
***

**Figure S7.** Proposed equivalent circuit model used to fit three-electrode EIS data of micro-structured FTO-coated glass substrates.





**Figure S8.** Transmittance spectra of the micro-structured FTO-coated glass substrates studied in this work. A commercial flat FTO-coated glass is used as the reference.





**Figure S9.** Pseudo sheet-resistance (labelled “Sheet resistance”, grey squares) and transmittance at a wavelength of 400 nm (λ = 400 nm, orange circles, extracted from Figure S8) of the micro-structured FTO-coated glass substrates studied in this work.


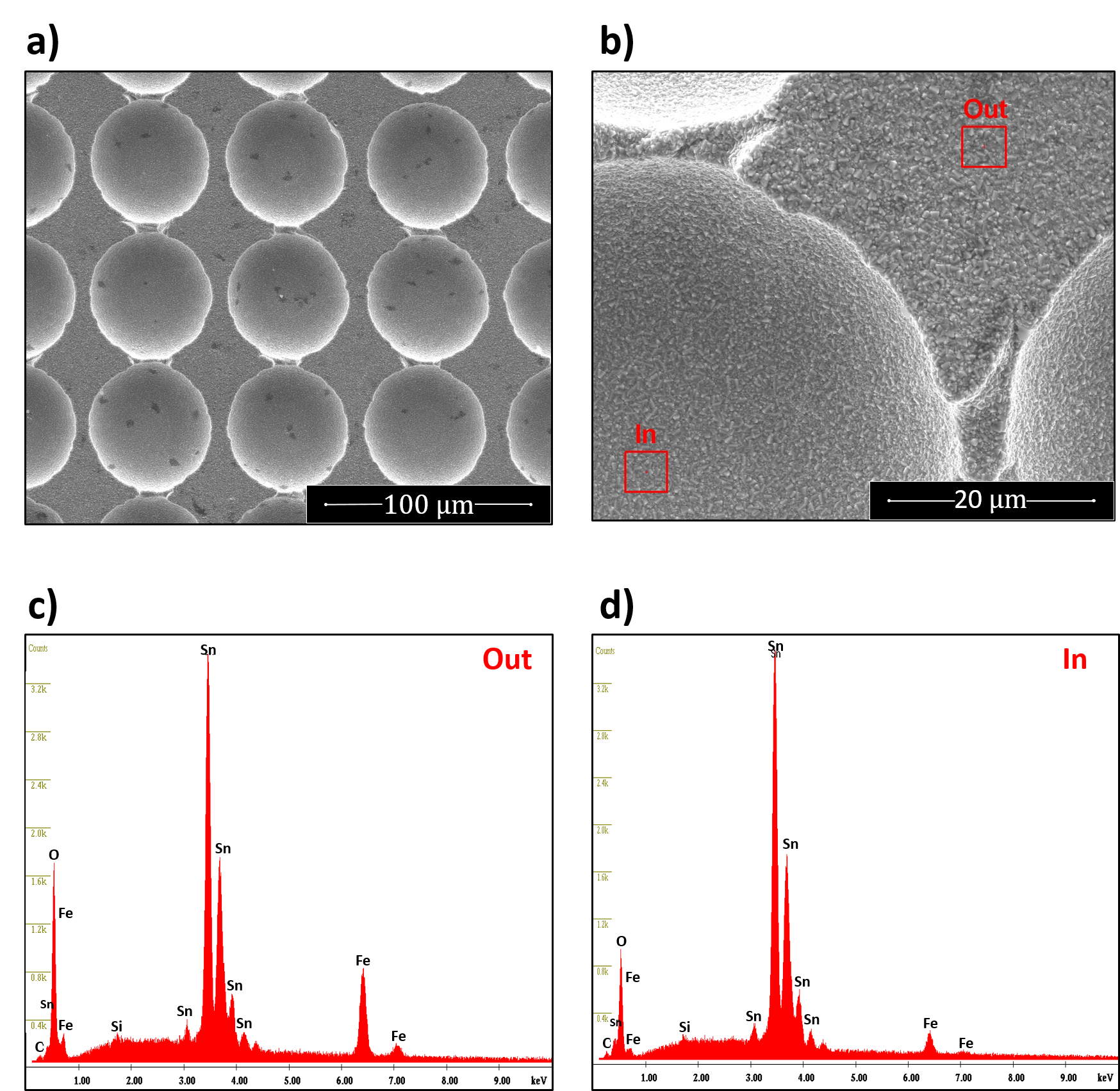


**Figure S10.** SEM images of a representative thin-film bare α-Fe_2_O_3_ photoelectrode prepared from a micro-structured FTO-coated glass substrate from the A-60-90 sample series (a and b). EDS analysis performed outside (c) and inside (d) of the micro-scale feature highlighted in b).

**S5. Additional information on the long-term chronoamperometry test**


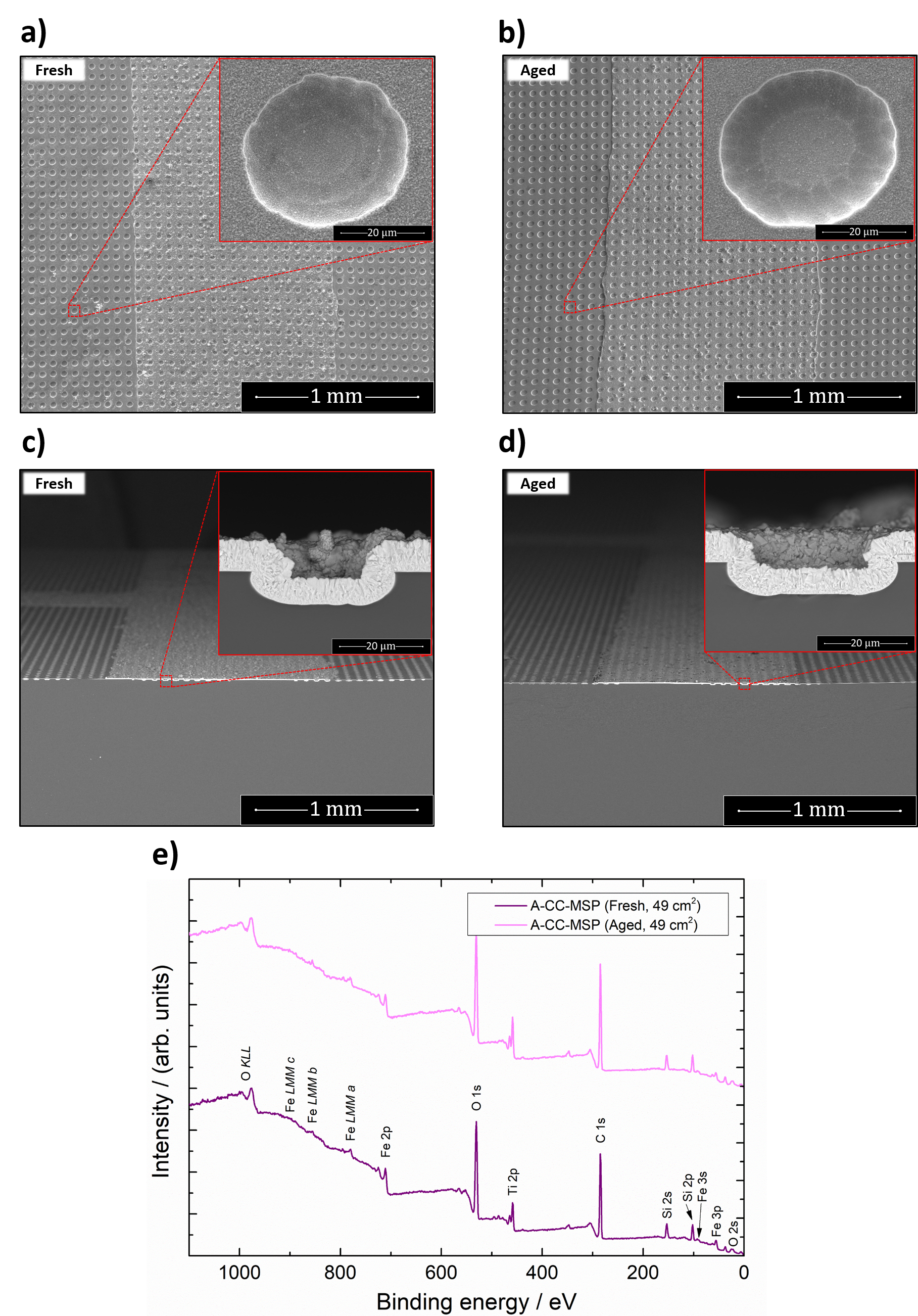


**Figure S11.** Surface morphology and elemental composition of fresh (left-side) and 1000 h aged photoelectrodes (right-side) of A-CC-MSP thin-film α-Fe_2_O_3_ photoelectrodes: top-view high-resolution SEM images (a and b, magnification of 100x); cross-section high-resolution SEM images (c and d, magnification of 100x and 5000x); wide-scan survey XPS spectra (e, detailed XPS regions for Fe 2p and O 1s can be found in Figure S12).


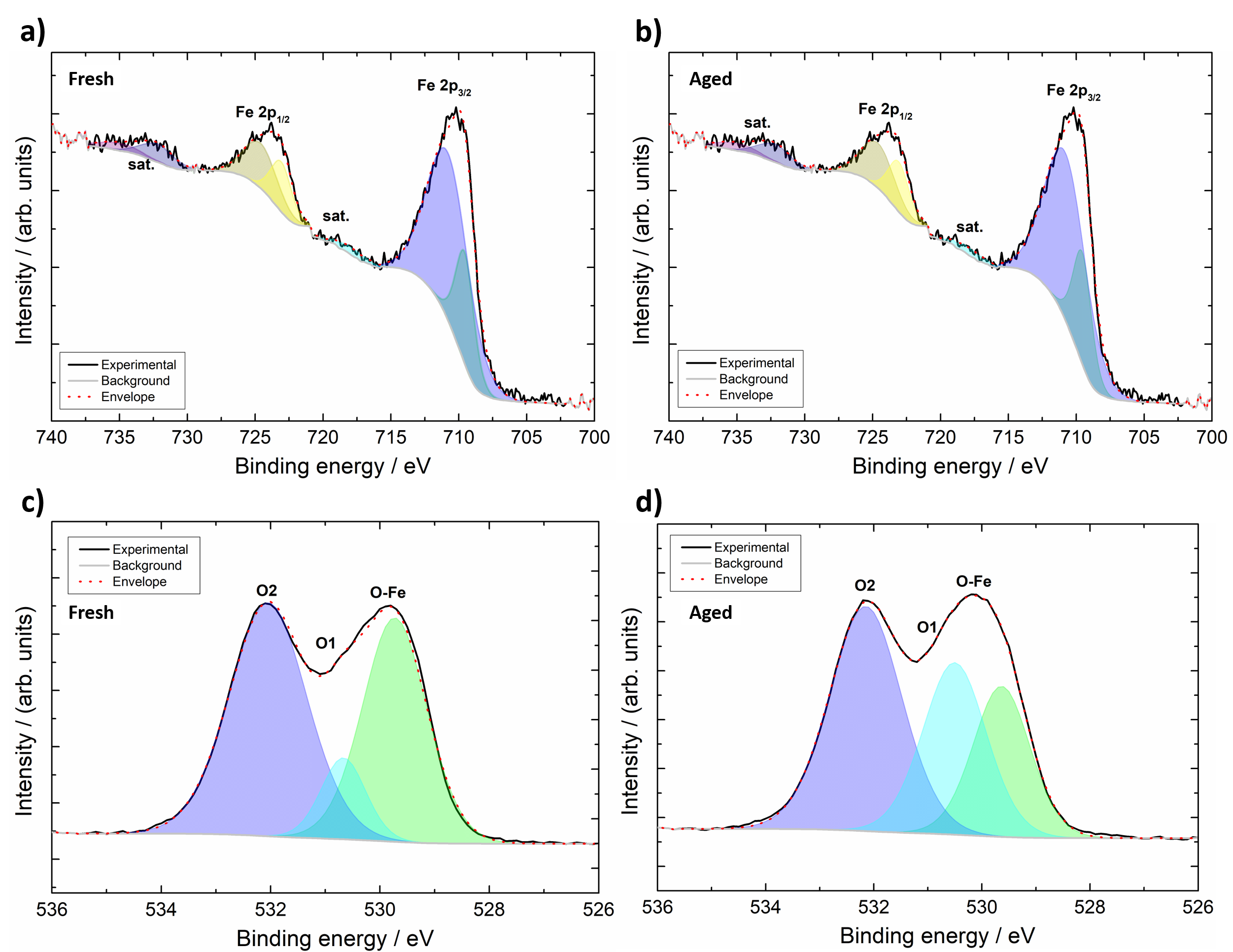


**Figure S12.** Detailed XPS regions for Fe 2p (a and b) and O 1s (c and d) for fresh and aged (after a 1000 h long chronoamperometry) thin-film bare α-Fe_2_O_3_ photoelectrodes prepared from optimized large-area micro-structured FTO-coated glass substrates with FTO current collectors.


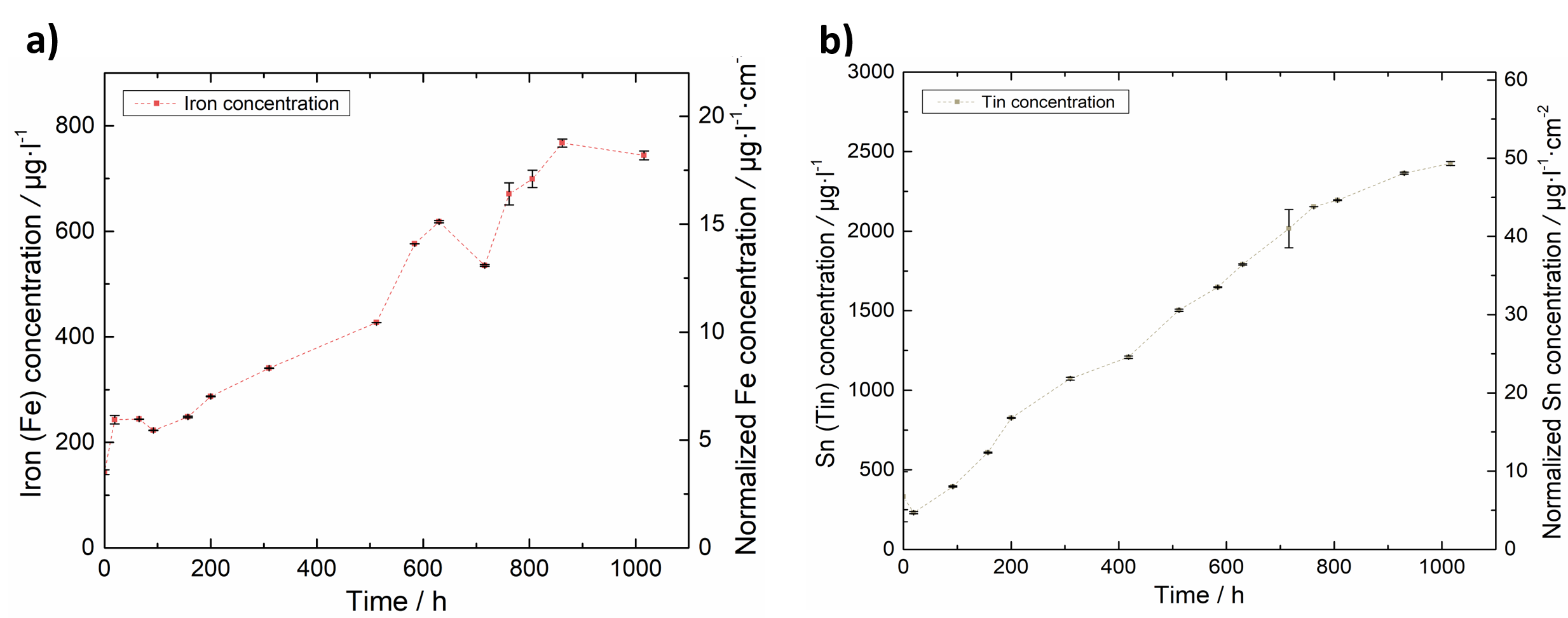


**Figure S13.** Mass concentration of iron (a) and tin (b) in the electrolyte (4 M KOH aqueous solution) used for the 1000 h long chronoamperometry test, as estimated by ICP-OES.

**Table S2.** Surface atomic composition of both fresh and aged thin-film bare α-Fe_2_O_3_ photoelectrodes prepared from optimized large-area micro-structured FTO-coated glass substrates with FTO current collectors. All values were estimated after peak-fitting the wide XPS spectrum in Figure S11e.

| **Sample** | **Orbital ID** | **Atomic composition / %** | **Fe/O atomic ratio** |
| --- | --- | --- | --- |
| **Fresh** thin-film α-Fe_2_O_3_ | C 1s | 24.66 | 0.32 |
|  | Fe 2p | 13.91 |  |
|  | Ti 2p | 13.95 |  |
|  | O 1s | 42.92 |  |
|  | Si 2p | 4.55 |  |
| **Aged (1000 h)** thin-film α-Fe_2_O_3_ | C 1s | 26.37 | 0.25 |
|  | Fe 2p | 10.75 |  |
|  | Ti 2p | 15.72 |  |
|  | O 1s | 43.03 |  |
|  | Si 2p | 4.14 |  |

**References**

[1] R. K. Tolod, S. Hernández, N. Russo, *Catalysts* **2017**, 7.

[2] X. Yao, D. Wang, X. Zhao, S. Ma, P. S. Bassi, G. Yang, W. Chen, Z. Chen, T. Sritharan, *Energy Technology* **2018**, 6, 100.

[3] H. Lu, V. Andrei, K. J. Jenkinson, A. Regoutz, N. Li, C. E. Creissen, A. E. H. Wheatley, H. Hao, E. Reisner, D. S. Wright, S. D. Pike, *Advanced Materials* **2018**, 30, 1804033.

[4] X. Zhu, X. Liang, P. Wang, B. Huang, Q. Zhang, X. Qin, X. Zhang, *Catalysis Today* **2020**, 340, 145.

[5] A. Qayum, M. Guo, J. Wei, S. Dong, X. Jiao, D. Chen, T. Wang, *Journal of Materials Chemistry A* **2020**, 8, 10989.

[6] I. Y. Ahmet, Y. Ma, J.-W. Jang, T. Henschel, B. Stannowski, T. Lopes, A. Vilanova, A. Mendes, F. F. Abdi, R. van de Krol, *Sustainable Energy & Fuels* **2019**, 3, 2366.

[7] M. Huang, W. Lei, M. Wang, S. Zhao, C. Li, M. Wang, H. Zhu, *Journal of Materials Chemistry A* **2020**, 8, 3845.

[8] V. Andrei, G. M. Ucoski, C. Pornrungroj, C. Uswachoke, Q. Wang, D. S. Achilleos, H. Kasap, K. P. Sokol, R. A. Jagt, H. Lu, T. Lawson, A. Wagner, S. D. Pike, D. S. Wright, R. L. Z. Hoye, J. L. MacManus-Driscoll, H. J. Joyce, R. H. Friend, E. Reisner, *Nature* **2022**, 608, 518.

[9] H. Cai, W. Zhao, G. Xiao, Y. Hu, X. Wu, H. Ni, S. Ikeda, Y. Ng, J. Tao, L. Zhao, F. Jiang, *Advanced Science* **2023**, 10, 2205726.

[10] P. Patil Kunturu, M. Lavorenti, S. Bera, H. Johnson, S. Kinge, M. C. M. van de Sanden, M. N. Tsampas, *ChemSusChem* **2024**, 17, e202300969.

[11] H. Lee, J.-H. Choi, K. Jung, H. Lim, D.-G. Choi, J.-Y. Jung, J.-H. Jeong, T. W. Kim, J. H. Park, J. Lee, *Journal of Industrial and Engineering Chemistry* **2023**, 125, 325.

[12] A. Singh, B. S. De, S. Karmakar, S. Basu, *ACS Applied Energy Materials* **2023**, 6, 4642.

[13] C. Murugan, A. S. Mary, A. Pandikumar, *Industrial & Engineering Chemistry Research* **2024**, 63, 4329.

[14] C. K. Ong, *Doctor of philosophy* Imperial College London, London, UK, January 2013, **2013**.

[15] J. Ihssen, A. Braun, G. Faccio, K. Gajda-Schrantz, L. Thöny-Meyer, *Current Protein & Peptide Science* **2014**, 15, 374.

[16] a) T. Lopes, P. Dias, L. Andrade, A. Mendes, *Solar Energy Materials and Solar Cells* **2014**, 128, 399; b) F. Le Formal, M. Grätzel, K. Sivula, *Advanced Functional Materials* **2010**, 20, 1099.

[17] A. Hankin, F. E. Bedoya-Lora, C. K. Ong, J. C. Alexander, F. Petter, G. H. Kelsall, *Energy & Environmental Science* **2017**, 10, 346.

[18] P. Dias, A. Vilanova, T. Lopes, L. Andrade, A. Mendes, *Nano Energy* **2016**, 23, 70.

[19] a) Photoelectrochemical Demonstrator Device for Solar Hydrogen Generation, <https://cordis.europa.eu/project/id/621252>, accessed: June 06, 2021; b) A. Vilanova, T. Lopes, C. Spenke, M. Wullenkord, A. Mendes, *Energy Storage Materials* **2018**, 13, 175.

[20] A. Vilanova, T. Lopes, A. Mendes, *Journal of Power Sources* **2018**, 398, 224.

[21] A. Landman, R. Halabi, P. Dias, H. Dotan, A. Mehlmann, G. E. Shter, M. Halabi, O. Naseraldeen, A. Mendes, G. S. Grader, A. Rothschild, *Joule* **2020**, 4, 448.

[22] A. Vilanova, P. Dias, J. Azevedo, M. Wullenkord, C. Spenke, T. Lopes, A. Mendes, *Journal of Power Sources* **2020**, 454, 227890.

[23] C. Lo Vecchio, G. Giacoppo, O. Barbera, A. Carbone, V. Baglio, A. S. Aricò, G. Monforte, S. Trocino, in *Catalysts*, Vol. 14, 2024.

[24] R. Gómez, presented at EuroNanoForum 2023, Lund, Sweden, 12 june 2023, **2023**.

[25] W. J. Lee, P. S. Shinde, G. H. Go, E. Ramasamy, *International Journal of Hydrogen Energy* **2011**, 36, 5262.

[26] P. S. Shinde, G. H. Go, W. J. Lee, *International Journal of Energy Research* **2013**, 37, 323.

[27] K. Walczak, Y. Chen, C. Karp, J. W. Beeman, M. Shaner, J. Spurgeon, I. D. Sharp, X. Amashukeli, W. West, J. Jin, N. S. Lewis, C. Xiang, *ChemSusChem* **2015**, 8, 544.

[28] S. Dilger, M. Trottmann, S. Pokrant, *ChemSusChem* **2019**, 12, 1931.

[29] S. Franz, H. Arab, G. L. Chiarello, M. Bestetti, E. Selli, *Advanced Energy Materials* **2020**, 10, 2000652.

[30] M.-K. Son, in *Energies*, Vol. 14, 2021.

[31] X. Xiao, M. Iwase, G. Yin, M. Nakabayashi, T. Higashi, N. Shibata, K. Domen, T. Watanabe, *ACS Sustainable Chemistry & Engineering* **2019**, 7, 19407.

[32] L.-J. Huang, G.-M. Zhang, Y. Zhang, B.-J. Li, N.-F. Ren, L. Zhao, Y.-L. Wang, *Acta Metallurgica Sinica (English Letters)* **2021**, 34, 973.

[33] H. Dotan, N. Mathews, T. Hisatomi, M. Gratzel, A. Rothschild, *J Phys Chem Lett* **2014**, 5, 3330.

[34] R. J. Gilliam, J. W. Graydon, D. W. Kirk, S. J. Thorpe, *International Journal of Hydrogen Energy* **2007**, 32, 359.

[35] M. Schalenbach, A. Zeradjanin, O. Kasian, S. Cherevko, K. Mayrhofer, *International Journal of Electrochemical Science* **2018**, 13, 1173.
